# Significant regional inequalities in the prevalence of intellectual disability and trends from 1990 to 2019: a systematic analysis of GBD 2019

**DOI:** 10.1017/S2045796022000701

**Published:** 2022-12-21

**Authors:** R. Nair, M. Chen, A. S. Dutt, L. Hagopian, A. Singh, M. Du

**Affiliations:** 1 Radboud University Medical Center, Radboud Institute for Health Sciences, Nijmegen, The Netherlands; 2 Department of Public Health Dentistry, Manipal Academy of Higher Education, Mangalore, Karnataka, India; 3 Office of Education Research, Nanyang Technological University, National Institute of Education, 1 Nanyang Walk, 637616 Singapore, Singapore; 4 Psychology, and Child and Human Development, Nanyang Technological University, National Institute of Education, 1 Nanyang Walk, 637616 Singapore, Singapore; 5 Johns Hopkins Medicine-Kennedy Krieger Institute, 707 North Broadway, Baltimore, Maryland 21205, USA; 6 Centre for Epidemiology and Biostatistics, Melbourne School of Population and Global Health, University of Melbourne, Melbourne, Victoria, Australia; 7 Cheeloo College of Medicine, Shandong University, Jinan, China

**Keywords:** GBD, inequalities, intellectual disability, prevalence

## Abstract

**Aims:**

Policymakers and researchers have little evidence on prevalence rates of intellectual disability (ID) or their changes over time to tailor healthcare interventions. Prevalence rates and trends of ID are especially lacking in regions with lower socio-demographic development. Additionally, the assessment of inequalities in the prevalence of ID across regions with varying socio-demographic development is understudied. This study assessed variations in prevalence rates of ID from 1990 to 2019 and the related inequalities between low and high socio-demographic index (SDI) regions.

**Methods:**

This study used global data from 1990 to 2019 for individuals with ID from the 2019 Global Burden of Diseases study. Data analyses were performed from September 2021 to January 2022. Prevalence for individuals with ID was extracted by sex, age groups and SDI regions. Annual percentage change (APC) was estimated for each demographic group within SDI regions to assess their prevalence trends over 30 years. Relative and absolute inequalities were calculated between low and high SDI regions for the various age groups.

**Results:**

In 2019, there were 107.62 million (1.74%) individuals with ID, with an APC of −0.80 (−0.88 to −0.72). There was a slightly higher prevalence among males (1.42%) than females (1.37%). The highest prevalence rates were found in the low-middle SDI regions (2.42%) and the lowest prevalence rates were in the high SDI regions (0.33%). There was a large reduction in the prevalence rate between the youngest age group *v*. the oldest age group in all the SDI regions and at all time points. The relative inequalities between low and high SDI regions increased over three decades.

**Conclusions:**

While an overall decrease in global prevalence rate for ID was found, relative inequalities continue to increase with lower SDI regions needing more comprehensive support services. The demographic trends indicate a significantly higher mortality rate among those with ID *v*. the rest of the population. Our study highlights the necessity for policies and interventions targeting lower SDI regions to mobilise resources that better support individuals with ID and achieve sustainable development goals proposed by the United Nations.

## Introduction

Intellectual disability (ID) is a life-long condition that impacts individuals in key areas of health, wellbeing, education, employment, citizenship, community participation and economic sustainability (Thompson *et al*., 2009; McKenzie *et al*., 2016; Scior *et al*., 2016; Reynolds *et al*., 2022). The American Association on Intellectual and Developmental Disabilities, Diagnostic Statistical Manual – 5th edition (DSM-5; 2013) and International Classification of Diseases – 10th edition (ICD-10) define ID as limitations in intellectual functioning and adaptive behaviour (World Health Organization, 1978; Schalock *et al*., 2010; American Psychiatric Association, 2013). Intellectual functioning refers to an individual's general mental capacity, such as learning, reasoning, problem-solving, etc. Adaptive behaviour is defined in terms of functioning in three areas of conceptual (e.g. language, numeracy and self-direction), social (e.g. social problem solving, interpersonal skills and social responsibility) and practical skills (e.g. personal care, vocational skills, community participation and healthcare) that are learned and performed by people in their everyday lives.

The DSM-5 and ICD-10 identify ID among individuals based on an intellectual quotient of less than 70 on cognitive test batteries and a below-average (or lower) adaptive functioning quotient reported by adaptive behaviour measures. Although these international organisations provide similar diagnostic frameworks for the identification of ID, changes in diagnostic practices (e.g. the tightening and broadening of the diagnostic criteria for ID) have been observed over the past few decades. These changes could have had an impact on prevalence estimates for ID over the years (Mckenzie *et al*., 2016). Despite changes in diagnostic practices, the identification of ID continues to inform service planning and allocation of resources through structural supports, interventions and accommodations. International goals for such efforts are outlined by the United Nations' sustainable development goals (SDGs) 2030 (United Nations-Department of Economic and Social Affairs Disability, 2018). SDGs 1, 2, 3, 4, 8, 10, 11 and 17 are directly applicable to individuals with ID (United Nations-Department of Economic and Social Affairs, 2019). These SDGs promote inclusive and equitable education and healthcare. The SDGs also promote employment, poverty alleviation, hunger eradication and provide social opportunities (United Nations-Department of Economic and Social Affairs Disability, 2018).

Several intervenable prenatal, perinatal and postnatal risk factors that interfere with normal brain development can result in ID among newborns (Huang *et al*., 2016; Nemerimana *et al*., 2018). Prenatal factors during pregnancy such as advanced parental age, exposure to alcohol and tobacco, chronic maternal illnesses (e.g. diabetes, hypertension, asthma, etc.), certain maternal infections (e.g. rubella, chickenpox, syphilis, etc.) and maternal malnutrition increases the risk for ID (Nemerimana *et al*., 2018). Perinatal factors such as birth complications, low birth weight, preterm birth and exposure to perinatal infections are other risk factors for the development of ID. Postnatal risk factors include lead and mercury poisoning, severe child malnutrition, central nervous system malignancies, etc. Additionally, inequalities in prenatal, perinatal and postnatal child developmental outcomes pertaining to ID for low- and middle-income countries (as per their gross national income) have been reported (Walker *et al*., 2011; de Graaf *et al*., 2013). Several of the risk factors that contribute to ID could be prevented by providing quality healthcare services during pregnancy, delivery and soon after birth to mothers and children (Sutherland *et al*., 2012). In addition, healthcare services are known to vary according to the individual-level per-capita income and country-level socio-economic position as measured using highest attained education (Stolk *et al*., 2016; Nusselder *et al*., 2021). As these healthcare needs may not be adequately met in several countries, primary studies on the distribution of ID and their meta-analyses indicate an increasing trend of prevalence of ID in low- and middle-income countries compared to high-income nations (Carulla *et al*., 2011; Maulik *et al*., 2011).

Studies published in the past decade on the worldwide prevalence of ID either have reported a descriptive range of rates estimated at 1–3% (Maulik *et al*., 2011; McKenzie *et al*., 2016) or have focused on a specific country (Arora *et al*., 2018), or certain age groups (Olusanya *et al*., 2018, 2020). ID has been associated with high mortality rates among the older populations, referred to as a mortality disadvantage (Landes, 2017; Reppermund and Walker, 2021). Similarly, several risk factors associated with ID, such as lack of optimal nutrition and inadequate access to child and maternal health services, are significant challenges faced by countries with lower socio-economic development (Graham, 2005; Simkiss *et al*., 2011; Banks *et al*., 2017). Therefore, there is a need to delineate detailed prevalence rates among individuals with ID across various age groups and countries of varying socio-economic development to inform national and global policies. These policies could target services to quantify better the disease burden and the resources required to address the needs and rights of individuals with ID as mandated by the SDGs. To this end, this paper aims to report worldwide trends in prevalence estimates and the annual percentage change (APC) for ID based on the Global Burden of Diseases (GBD) 2019 study (Vos *et al*., 2020), across sex, age, socio-economic development (i.e. low, low-middle, middle, high-middle and high) and for all regions listed in the GBD 2019 study across 30 years (1990–2019).

## Methods

### Data source

Individuals with ID and related conditions (i.e. ICD-10 codes F70–79 or ICD-9 codes 317–319) were included in this study (World Health Organization, 1993). While the GBD 2019 study (Vos *et al*., 2020) provides details on the methodology, some relevant details about the data sources and data processing for ID are presented here. Those with ID were added to the dataset based on ICD-10 and legacy ICD-9 codes (World Health Organization, 1978, 1993). The prevalence rates for ID were calculated from IQ survey data, cognitive function data, international educational attainment data, relevant registries and other survey data (Vos *et al*., 2020). They combine data from standardised tests for IQ from various sources using analytical methods described earlier. Data were then modelled using Disease Modelling – Meta-regression (Dismod-MR 2.1) and distribution fitted using mean and prevalence. Uncertainty intervals (UI) were calculated based on 1000 draws of simulations. This expressed the relevant uncertainty from random probability distributions of input sources, variations in sex, age, diagnostic uncertainties, data manipulations and choice of models used in these estimations.

### Socio-demographic information

This study sourced data for annual prevalence rates and estimated the number of individuals with ID for specific age groups, sexes and socio-demographic index (SDI) from 1990 to 2019 based on the GBD 2019 study (Vos *et al*., 2020). Separate information was extracted for both females and males for their global prevalence rates and numbers. Global prevalence rates and numbers were separately extracted for the five age groups that included 0–9, 10–24, 25–49, 50–69 and 70+ years. Regions listed in the GBD 2019 study were grouped according to their SDI levels in 2019. SDI is a composite made of several variables that predict a region's social and economic conditions (Global Burden of Diseases Collaborative Network, 2020). These socio-economic conditions play a key role in predicting the health of the relevant regions. Thus, the values for low, low-middle, middle, high-middle and high SDI regions were extracted. All regions were also separately extracted for a more detailed perspective of their prevalence rates. The results across each region are attached in the online Supplementary Appendix.

### Data analyses

This study complies with the Guidelines for Accurate and Transparent Health Estimates Reporting (GATHER) statement (Stevens *et al*., 2016). Global prevalence rates of ID are presented in the tables as represented in a per 100 000 of the population (as a standard practice of reporting prevalence in epidemiological studies) and in text in the Results section as a percentage or per 100 of the population (by dividing the prevalence rate per 100 000 by a 1000) for ease of reading. Similarly, the number of individuals is presented in the tables as *n* × 1000 and in millions (by dividing the number by 1 000 000) in the text within the Results section for each category. Trends in prevalence rates over the three decades (i.e. one time point for each year) of this study were assessed by calculating the APC for the overall global population and each age range within the SDI regions separately. The prevalence rate, age and SDI were extracted for the APC calculations. Following previously used methodology, models were fitted for each row where the prevalence rates were regressed against the year (Kim *et al*., 2000). The (e*^β^* − 1) × 100 indicates the APC, and the (e*^β^*^±1.96s.e.^ − 1) × 100 indicates the 95% confidence intervals (CIs) of the APC, where *β* and s.e. denote the coefficient and standard error. APC was considered statistically significant, where the 95% CI did not include a zero. A positive APC indicates an increasing trend, whereas a negative APC indicates a decreasing trend. Consistent with recommended practices for inequality estimation (Mackenbach *et al*., 2016), absolute and relative inequalities were calculated by comparing the average prevalence rates of the higher SDI regions (high and high-middle SDI) with lower SDI regions (low and low-middle SDI). Absolute inequalities were calculated as a difference of the average prevalence rates for each year of the higher SDI regions from the lower SDI regions. Similarly, the relative inequalities were calculated as a ratio of the average prevalence rates for each year of the higher SDI *v*. lower SDI regions. Further trends in absolute and relative inequalities in prevalence rates were mapped across the years. Global heat maps for prevalence rates and APCs were also created. All analyses were carried out using R-studio (version 1.2.1335).

## Results

### ID prevalence by sex, age and SDI

Globally, 92.8 million individuals or approximately 1.74% of the population were estimated to have ID in 1990 (Table 1). There was a steady increasing trend in the global number of individuals through 2019, with an estimated 107.6 million individuals with ID. But the proportion of individuals with ID continued to decrease to 1.39% of the global population in 2019, with an APC of −0.80 (−0.88 to −0.72). There were slightly higher estimates for the number and proportion of males *v*. females with ID. The 2019 data indicate 54.9 million males (1.42%) *v*. 52.7 million females with ID (1.37%).

**Table 1.** Prevalence (i.e. number and rate) for ID

Variables	1990		2000		2010		2019	
	Prevalence no. (95% UI) × 10^3^	Prevalence rate (95% UI)^a^	Prevalence no. (95% UI) × 10^3^	Prevalence rate (95% UI)^a^	Prevalence no. (95% UI) × 10^3^	Prevalence rate (95% UI)^a^	Prevalence no. (95% UI) × 10^3^	Prevalence rate (95% UI)^a^
Global	92 835.53 (58 341.00–128 614.52)	*1735.29* (*1090.52–2404.08)*	103 780.49 (65 769.93–143 298.61)	1686.00 (1068.49–2328.01)	108 039.79 (67 513.65–149 970.67)	1546.23 (966.23–2146.33)	107 619.59 (65 821.82–150 394.04)	1390.89 (850.69–1943.71)
Sex								
Female	45 174.43 (29 181.81–61 648.94)	*1700.78 (1098.67–2321.03)*	50 347.64 (32 643.00–68 814.67)	1648.71 (1068.95–2253.44)	52 704.47 (33 675.58–72 106.72)	1516.41 (968.91–2074.65)	52 673.63 (33 124.00–72 814.04)	1365.84 (858.92–1888.09)
Male	47 661.10 (29 379.87–66 742.44)	*1769.32 (1090.67–2477.68)*	53 432.86 (33 065.11–74 583.40)	1722.72 (1066.05–2404.63)	55 335.33 (33 839.95–77 648.71)	1575.73 (963.63–2211.13)	54 945.96 (32 836.69–77 631.53)	1415.78 (846.09–2000.31)
Age								
0–9 years	29 003.70 (18 562.60–39 660.25)	2382.67 (1524.93–3258.11)	29 815.24 (19 248.36–40 696.11)	*2417.06 (1560.42–3299.15)*	28 690.09 (18 278.11–39 342.54)	2253.41 (1435.62–3090.09)	25 792.62 (15 949.95–35 870.99)	1957.63 (1210.58–2722.56)
10–24 years	31 299.17 (19 963.21–42 885.63)	2020.65 (1288.81–2768.66)	35 246.65 (22 759.82–48 223.75)	*2053.34 (1325.90–2809.33)*	36 280.14 (23 116.80–49 692.63)	1968.58 (1254.57–2696.87)	35 349.20 (22 324.66–48 753.54)	1898.58 (1199.04–2618.52)
25–49 years	25 102.10 (15 506.07–35 018.25)	1476.67 (912.17–2060.00)	30 112.93 (18 750.27–41 963.23)	1415.90 (881.63–1973.09)	32 715.28 (20 169.14–45 755.53)	1336.03 (823.67–1868.57)	34 330.69 (20 847.21–48 236.56)	1264.29 (767.73–1776.39)
50–69 years	6452.46 (3823.23–9229.84)	*945.92 (560.48–1353.07)*	7321.81 (4325.45–10 514.48)	905.91 (535.18–1300.94)	8750.10 (5075.87–12 608.11)	822.41 (477.07–1185.02)	10 229.08 (5792.90–14 905.95)	741.80 (420.10–1080.96)
70+ years	978.10 (550.83–1454.20)	*485.30 (273.30–721.52)*	1283.86 (722.39–1893.35)	474.94 (267.24–700.41)	1604.18 (901.37–2370.22)	447.00 (251.16–660.46)	1917.99 (1076.46–2837.04)	413.65 (232.16–611.86)
Socio-demographic index								
Low	13 835.45 (8975.39–18 927.64)	2619.65 (1699.43–3583.83)	19 626.62 (12 837.52–26 691.19)	*2854.66 (1867.19–3882.19)*	24 103.84 (15 716.43–32 775.46)	2674.63 (1743.94–3636.86)	26 361.13 (16 753.87–36 249.27)	2335.58 (1484.38–3211.66)
Low-middle	38 175.51 (25 804.98–51 066.11)	*3379.42 (2284.34–4520.53)*	41 733.16 (28 217.30–55 766.47)	3103.67 (2098.50–4147.32)	43 229.83 (28 779.94–58 070.85)	2759.42 (1837.06–3706.74)	42 684.17 (28 037.13–57 676.42)	2419.76 (1589.42–3269.67)
Middle	27 439.90 (17 127.08–37 929.91)	*1598.36 (997.64–2209.39)*	28 712.70 (18 089.15–39 796.63)	1450.72 (913.96–2010.74)	28 322.21 (17 691.64–39 685.82)	1284.95 (802.65–1800.51)	26 953.72 (16 266.69–38 175.96)	1124.68 (678.75–1592.94)
High-middle	9568.14 (5171.76–14 104.60)	*831.70 (449.55–1226.03)*	10 021.71 (5453.729–14 762.27)	796.88 (433.66–1173.83)	8907.52 (4627.55–13 344.13)	657.00 (341.32–984.24)	8266.41 (4099.05–12 615.88)	577.91 (286.57–881.98)
High	3781.26 (1388.49–6271.12)	*460.00 (168.91–762.90)*	3645.19 (1316.88–6091.34)	412.83 (149.14–689.86)	3431.84 (1139.29–5847.37)	359.16 (119.23–611.96)	3311.45 (1055.76–5746.42)	326.77 (104.18–567.05)

UI, uncertainty intervals.

a Prevalence rates are per 100 000.

A considerable reduction was observed in the number (and prevalence rate) of individuals with ID across the increasing age categories. In 1990 only 0.98 million individuals (0.49% of the population) had ID among those aged 70+ years compared to 29 million (2.38% of the population) in the 0–9 years age group. Similarly, in 2019 there were only 1.92 million individuals with ID in the 70+ years age group (0.41% of the population) when compared to 25.8 million (1.98% of the population) in the 0–9 years age group.

**Fig. 1.**
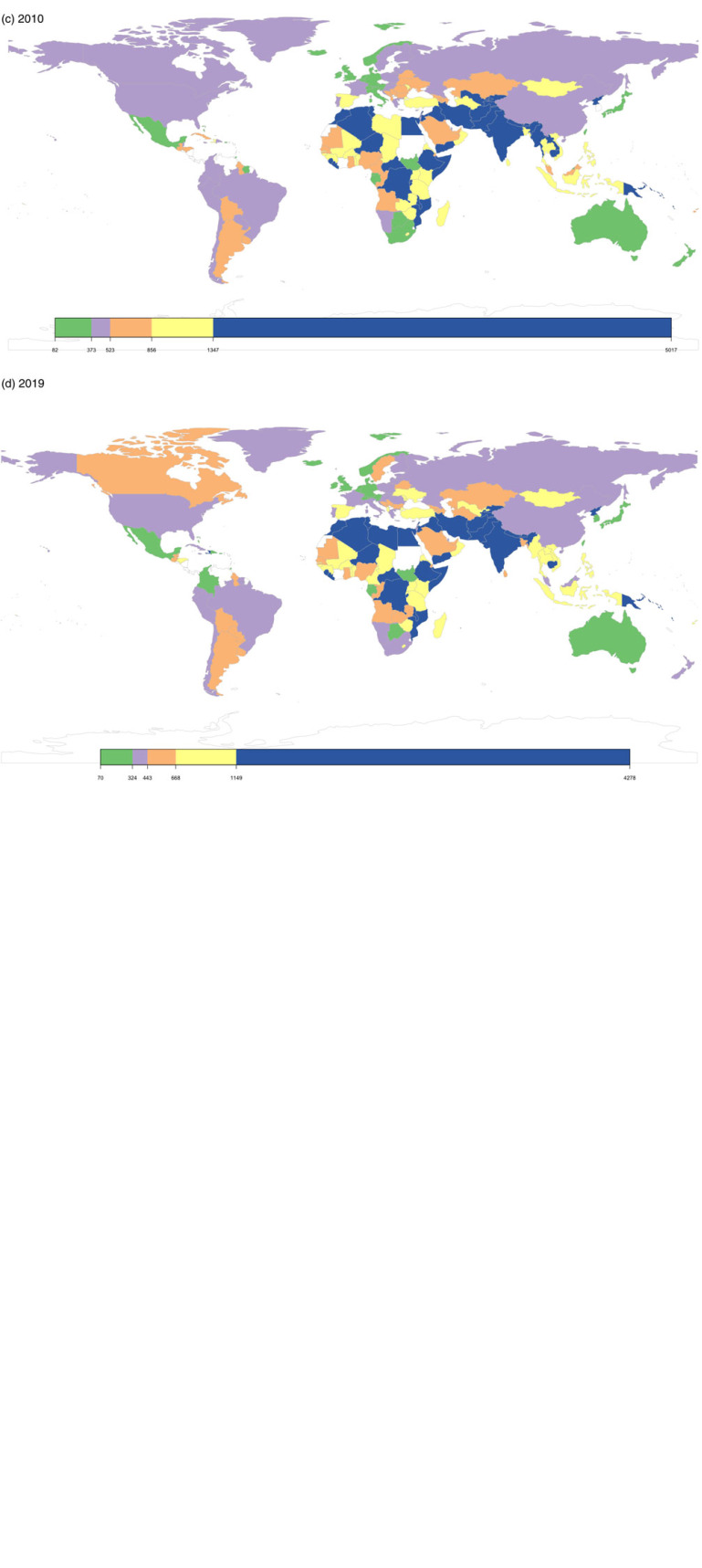
Global prevalence of ID by country and territory: (a) 1990; (b) 2000; (c) 2010 and (d) 2019.

In 1990, there was a relatively higher number and prevalence rate (38.2 million and 3.38%) of individuals with ID in the low-middle SDI regions compared to the low SDI (13.8 million and 2.62%, respectively), middle SDI (27.43 million and 1.60%), high-middle SDI (9.57 million and 0.83%) and high SDI (3.78 million and 0.46%) regions. This was true for 2000, 2010 and 2019, where the highest number and proportion of individuals with ID were in the low-middle SDI regions. The global distribution of the prevalence rate of ID (Fig. 1) shows the regional skews with regions in Asia and Africa skewing towards higher prevalence rates. Detailed country-specific values for prevalence rate in 2019 and APC for 30 years are given in the online Supplementary Appendix.

### ID prevalence in each SDI stratified by age

Table 2 shows the number of individuals with ID in the five SDI regions separated by the five age groups in 1990, 2000, 2010 and 2019. In the low SDI regions, the 0–9 years age group had the highest number in 2010 (8.67 million), while in all other age groups, the highest number was observed in 2019. In the low-middle SDI regions, the highest number aged 0–9 years were found in 1990 and 2000 with 13.04 million individuals in both those time points. The highest number in the 10–24 years age group was found in 2010 with 15.16 million individuals. For the three older age groups, the highest number of individuals were all found in 2019.

**Table 2.** Number of individuals and APC in prevalence rates by SDI and age

Variables	1990	2000	2010	2019	APC
	Number (95% UI) × 10^3^	Number (95% UI) × 10^3^	Number (95% UI) × 10^3^	Number (95% UI) × 10^3^	(1990–2019)
Low SDI					
0–9 years	5384.01 (3494.55–7334.94)	7431.77 (4880.29–10 074.89)	8669.39 (5648.68–11 813.85)	8430.26 (5324.79–11 609.20)	−0.70 (−0.91 to −0.48)
10–24 years	4650.02 (3053.45–6317.88)	6761.74 (4473.51–9151.62)	8432.33 (5549.95–11 418.67)	9686.02 (6213.90–13 220.82)	−0.37 (−0.54 to −0.19)
25–49 years	3051.85 (1964.51–4192.07)	4421.53 (2858.13–6071.35)	5652.71 (3652.77–7730.39)	6579.96 (4188.90–9092.43)	−0.34 (−0.51 to −0.17)
50–69 years	673.92 (418.92–955.18)	895.77 (559.48–1258.38)	1180.18 (746.56–1639.39)	1435.58 (895.98–2001.95)	0.01 (−0.18 to 0.19)
70+ years	75.64 (45.38–112.12)	115.80 (70.69–169.11)	169.22 (104.81–242.98)	229.32 (142.52–324.47)	0.97 (0.84–1.10)
Low-middle SDI					
0–9 years	13 046.31 (8902.36–17 359.14)	13 042.32 (8878.93–17 325.63)	12 011.98 (8017.60–16 036.29)	10 270.17 (6764.48–13 828.52)	−1.11 (−1.18 to −1.04)
10–24 years	12 946.28 (8803.99–17 245.09)	14 457.71 (9884.79–19 235.48)	15 157.57 (10 165.98–20 266.83)	14 789.20 (9851.74–19 875.94)	−0.82 (−0.87 to −0.78)
25–49 years	9581.54 (6444.45–12 887.17)	11 259.91 (7561.26–15 128.57)	12 405.55 (8198.84–16 725.20)	13 317.00 (8727.09–18 013.30)	−1.08 (−1.10 to −1.05)
50–69 years	2136.97 (1513.62–3145.06)	2595.22 (1709.52–3552.97)	3124.65 (2017.84–4260.99)	3647.44 (2334.97–5013.36)	−1.08 (−1.13 to −1.02)
70+ years	284.41 (174.18–402.68)	377.99 (238.34–532.65)	530.08 (334.88–740.71)	660.36 (420.54–918.48)	−0.47 (−0.54 to −0.40)
Middle SDI					
0–9 years	7797.20 (4915.28–10 750.74)	6975.84 (4356.03–9667.52)	6081.70 (3752.01–8505.27)	5373.21 (3183.83–7616.92)	−0.96 (−1.05 to −0.88)
10–24 years	9657.31 (6107.50–13 246.78)	9943.84 (6365.66–13 661.67)	9290.16 (5908.07–12 839.79)	8046.11 (4996.91–11 247.50)	−0.73 (−0.77 to −0.69)
25–49 years	7849.74 (4912.16–10 848.22)	9326.34 (5864.49–13 001.01)	9891.20 (6211.13–13 895.10)	9862.05 (5993.61–13 924.97)	−0.94 (−0.97 to −0.91)
50–69 years	1881.46 (1141.70–2648.77)	2128.69 (1283.32–3020.57)	2625.16 (1551.03–3770.00)	3158.14 (1787.80–4597.90)	−1.31 (−1.35 to −1.27)
70+ years	254.18 (144.97–372.30)	337.99 (193.62–493.80)	433.99 (249.57–631.75)	514.22 (288.86–759.80)	−1.08 (−1.16 to −1.01)
High-middle SDI					
0–9 years	2168.79 (1178.87–3184.65)	1822.68 (1014.09–2676.76)	1457.19 (765.63–2175.54)	1291.69 (636.98–1980.57)	−1.11 (−1.28 to −0.95)
10–24 years	3030.98 (1693.38–4415.76)	3162.20 (1790.08–4590.52)	2552.00 (1388.62–3762.11)	2067.14 (1089.01–3085.02)	−0.93 (−1.02 to −0.83)
25–49 years	3167.31 (1699.37–4682.73)	3682.21 (1986.04–5453.92)	3453.34 (1791.66–5183.07)	3305.21 (1646.51–5040.13)	−1.13 (−1.21 to −1.05)
50–69 years	1008.98 (514.55–1527.35)	1096.70 (559.39–1657.02)	1165.38 (549.49–1804.48)	1300.63 (584.11–2062.66)	−1.38 (−1.52 to −1.24)
70+ years	192.09 (100.88–290.71)	257.92 (133.32–393.14)	279.60 (140.98–432.36)	301.74 (146.51–473.31)	−1.25 (−1.38 to −1.12)
High SDI					
0–9 years	596.64 (185.26–1033.17)	530.90 (159.89–930.67)	457.95 (123.32–816.90)	416.26 (104.77–758.03)	−0.96 (−0.98 to −0.94)
10–24 years	1002.20 (365.81–1658.28)	906.98 (328.88–1508.03)	833.04 (273.84–1417.71)	746.90 (232.22–1290.50)	−0.87 (−0.89 to −0.84)
25–49 years	1442.25 (531.27–2379.61)	1411.01 (508.57–2348.59)	1298.84 (432.47–2216.83)	1253.05 (401.52–2157.99)	−0.98 (−1.02 to −0.94)
50–69 years	568.80 (224.74–926.67)	602.68 (225.72–1001.74)	651.36 (218.87–1103.29)	683.56 (228.68–1172.41)	−1.07 (−1.10 to −1.04)
70+ years	171.37 (80.52–271.32)	193.62 (86.15–310.73)	190.65 (75.79–319.36)	211.68 (79.43–362.69)	−1.69 (−1.77 to −1.61)

**Fig. 2.**
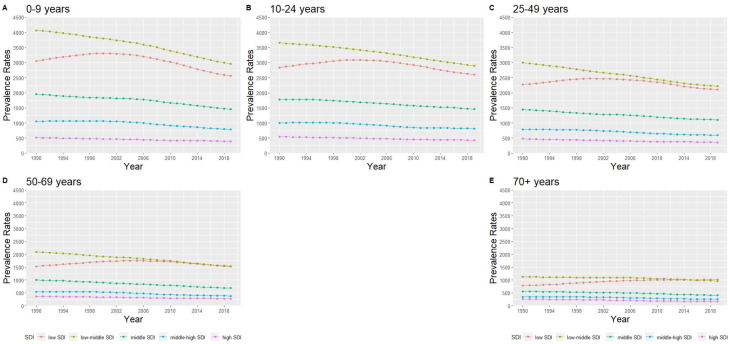
Trends of prevalence rates of the SDI regions in the five age groups: (a) 0–9, (b) 10–24, (c) 25–49, (d) 50–69 and (e) 70+ years.

The prevalence rates for all SDI regions by the five age groups (Fig. 2) show an overall decreasing trend over three decades. For all the age groups, the low-middle SDI and low SDI regions had the highest prevalence rates while the other regions were ordered with the next highest being the middle SDI regions, followed by the high-middle SDI region and the high SDI regions. Low SDI regions had an increasing trend until 2004 for the 0–69 year age groups, while the 70+ year age group had an overall increasing trend.

Among the low SDI regions, the APC values were negative for individuals with ID aged 0–9 years, 10–24 years and 25–49 years (Table 2). The APC value was close to 0 for individuals aged 50–69 years, with a CI including 0. In addition, for individuals aged 70 years and above, the APC value of 0.97 indicates a significantly increasing trend. For the other four SDI regions (low-middle, middle, high-middle and high SDI) over the 30 years, the APC values were all negative, and their CIs did not include the value of 0 for all age groups, indicating a decreasing trend of prevalence rates for ID in each age group.

**Fig. 3.**
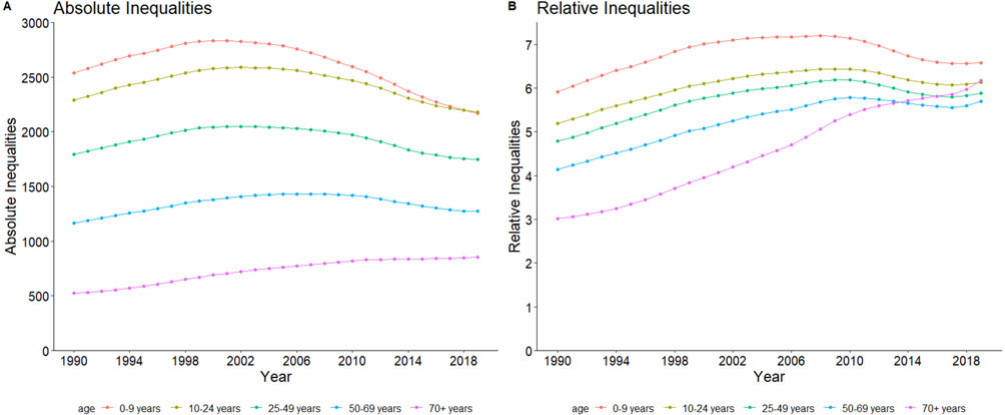
Trend of relative and absolute inequalities between high and low SDI regions for the five age groups: (a) absolute inequalities and (b) relative inequalities. *Note*: AI, absolute inequality as measured by difference between prevalence rate of high and low SDI regions; PR, prevalence ratio as measured by the ratio between the prevalence in high and low SDI regions.

The absolute inequalities estimate between low and high SDI regions showed marked differences according to age groups, with differences higher among younger age groups than older age groups across the three decades (Fig. 3). The 50–69 and 70+ years age groups had increasing absolute inequalities, while the other age groups had a decreasing trend. On the relative scale, while inequalities between low and high SDI regions increased for all age-groups, there was marked increase from three-fold to over five-fold among the 70+ years age group over the three decades.

## Discussion

This study quantified the global trends in the prevalence of individuals with ID. In 2019, an estimated 107.62 million individuals with ID formed about 2% of the global population. While there was a global trend of increasing number of individuals with ID, the prevalence rate continued to decrease across the three decades in this study. Consequently, absolute inequalities reduced or remained largely stagnant over the three decades. However, relative inequalities increased suggesting the need for more targeted services for people with ID in low SDI regions particularly in older age groups. The most notable findings were that regions with less socio-economic development had a far greater prevalence rate of ID, and the prevalence rate of ID reduced with age.

There was a slightly higher number and prevalence rate for males *v*. females, and it agrees with earlier studies that assessed the prevalence rates of individuals with ID (Boyle *et al*., 2011; McKenzie *et al*., 2016). The global data for the number of individuals with ID separated into age groups show that the younger ages had passed peak numbers in 2000 or 2010. Among the 25+ years age group, the numbers continued to rise in the three decades through 2019. This suggests a demographic shift in the number of individuals with ID, where the population overall gets older due to lower number of children globally being diagnosed with ID over time. This aligns with global population trends, where the number of individuals with ID follows the overall declining fertility rates (Pantazis and Clark, 2018; Da Silva *et al*., 2021). The more alarming trend in the age groups is the precipitous decrease in prevalence rates with increasing age. This suggests a significantly higher mortality over the lifespan among those with ID *v*. others (Glover *et al*., 2017; O'Leary *et al*., 2018; Cooper *et al*., 2020; Das-Munshi *et al*., 2021; Doyle *et al*., 2021). There was almost a five-fold reduction in the 70+ year old category *v*. the 0–9 year old category. An earlier systematic review found higher mortality rates among individuals with ID (Landes, 2017; O'Leary *et al*., 2018; Reppermund and Walker, 2021). These included studies were restricted to high SDI countries due to the absence of studies in other regions.

The analyses by SDI categories found a significant difference in the prevalence rates of ID which, was 5–6 times higher in the low- and low-middle income regions *v*. the high SDI regions. This increase was seen across time, where the more developed regions had progressively lower prevalence rates. Comparisons across time points indicate that the low SDI regions had the highest overall number of individuals with ID in 2019. Other SDI regions had their highest numbers in progressively earlier time periods, with the high SDI group peaking at or earlier than 1990. This large difference in rates could be attributable to the increased exposure to risk factors that include a lack of maternal and child health care, malnutrition, more births with proportionally higher genetic illnesses, etc. (Graham, 2005; Maulik *et al*., 2011; Simkiss *et al*., 2011; Banks *et al*., 2017). Thus, there are structural inequalities based on the socio-economic development of regions that predispose a greater number of individuals to ID in the lower SDI regions. The trends of overall increases in relative inequalities show the widening gap between the high and low SDI regions, and the relatively unchanged or increasing absolute inequalities emphasise the need for greater actions needed for care of those with ID in the lower SDI regions. These findings necessitate greater levels of resource-intensive care to assist with activities of daily living for individuals with ID in regions with a higher prevalence of ID (Global Burden of Diseases Collaborative, 2017).

The study found a global trend of decreasing prevalence rates of ID for all age groups except for the 50+ year groups in the low SDI regions. It is important to note that while all regions had similar APC reductions for the younger age groups, the trend across the age ranges suggests a pattern of greater reductions for the older age groups. This trend could be due to systemic changes in longevity that happened earlier in the higher SDI regions, followed later by the lower SDI regions (The Global Burden of Disease Child and Adolescent Health Collaboration, 2019).

### Limitations

The wide UI ranges across all regions suggest a scarcity of worldwide data on the prevalence of ID (Eurostat, 2015; Friedman *et al*., 2018). Although this study includes data from three decades, the direct comparison of the 70+ year group *v*. the youngest should be taken with caution as they are not the same cohort at different times. But the trend of decreasing prevalence of ID in all age groups suggests that this may be a worst-case scenario, as the current younger cohort possibly has a lower prevalence rate than the corresponding 70+ year group at their corresponding younger age.

### Implications

The worldwide higher mortality among those with ID calls for global action to help achieve SDGs specifically on providing equitable health and wellbeing for all by 2030. This finding suggests the need for considerable initiatives for preparing global healthcare systems to cater to the needs of those with ID. The inequalities estimated within this study highlight services for people with ID need to be scaled according to regional disadvantage, otherwise inequalities in social and health outcomes will escalate over time. This is especially true since many of these deaths are due to preventable causes which include respiratory and cardiovascular conditions (O'Leary *et al*., 2018).

The progressively greater prevalence of ID among lower SDI regions suggests a higher propensity for these regions to have risk factors associated with ID. As risk factors range from prenatal, perinatal and postnatal factors, it is important to concentrate efforts to provide maternal support to help avert these risk factors for ID. This is especially important in the lower SDI regions, especially South Asia, where the prevalence of ID is very high. Thus, there is a need for quantifying the causal variables to calculate the attributable risk of ID to produce policies and interventions that reduce the cases of preventable ID. This could contribute towards a greater equity between various regions in the world.

## Conclusions

In conclusion, our investigation provides detailed estimates of the prevalence of ID across different regions over three decades. The study found a decreasing trend of prevalence rates of ID, despite an increased number of individuals diagnosed with ID primarily observed in the low and low-middle SDI regions. Remarkable differences in ID prevalence rates and trends found between higher and lower SDI areas show significant inequities. Similarly, significant decreases in the prevalence rates among the older age groups suggest the lack of comprehensive and tailored healthcare provision both within and outside of clinical settings.

## Data Availability

All data used in this paper came from openly available dataset hosted by Global Burden of Diseases collaborative. The code for analyses and detailed extraction from the data set are given in detail in the online Supplementary Appendices.

## Supplementary material

For supplementary material accompanying this paper visit http://doi.org/10.1017/S2045796022000701. click here to view supplementary material
