# Supplementary material for: Significant regional inequalities in the prevalence of intellectual disability and trends from 1990 to 2019: a systematic analysis of GBD 2019
Source: Epidemiol Psychiatr Sci. 2022 Dec 21;31:e91. doi: 10.1017/S2045796022000701 (PMC9805697; doi:10.1017/S2045796022000701)
Supplement: Supplementary file 1 [file epssup.zip › S2045796022000701sup001.docx]

Appendix 1

**Table 1: Prevalence by Countries at Four Time Points (1990, 2000, 2010, 2019)**

|  | **1990** | | **2000** | | **2010** | | **2019** | |
| --- | --- | --- | --- | --- | --- | --- | --- | --- |
|  | ***N (10^3^)*** | **rate** | ***N (10^3^)*** | **rate** | ***N (10^3^)*** | **rate** | **N *(10^3^)*** | **rate** |
| Afghanistan | 450.79 (303.30 to 598.94) | 3947.79 (2656.12 to 5245.14) | 1143.42 (792.49 to 1517.01) | 5875.36 (4072.15 to 7795.06) | 1435.31 (983.20 to 1899.35) | 5016.78 (3436.53 to 6638.73) | 1637.62 (1118.02 to 2164.74) | 4278.29 (2920.83 to 5655.39) |
| Albania | 39.64 (23.11 to 56.34) | 1197.59 (698.35 to 1702.23) | 38.39 (22.99 to 54.43) | 1202.83 (720.45 to 1705.43) | 24.58 (14.14 to 35.30) | 850.95 (489.67 to 1222.21) | 18.23  (9.51 to 27.03) | 670.02 (349.55 to 993.61) |
| Algeria | 528.95 (348.91 to 715.51) | 2092.13 (1380.05 to 2830.03) | 667.95 (445.87 to 900.75) | 2151.40 (1436.09 to 2901.23) | 651.23 (419.34 to 885.71) | 1797.09 (1157.19 to 2444.15) | 665.76 (413.90 to 925.93) | 1590.93 (989.09 to 2212.63) |
| American Samoa | 0.21 (0.08 to 0.33) | 428.40 (170.81 to 686.04) | 0.28  (0.11 to 0.44) | 470.86 (194.30 to 746.52) | 0.27  (0.12 to 0.43) | 474.67 (203.77 to 753.15) | 0.27  (0.12 to 0.43) | 491.74 (216.87 to 776.29) |
| Andorra | 0.15  (0.04 to 0.27) | 275.34  (70.67 to 497.79) | 0.18  (0.04 to 0.32) | 269.76  (68.74 to 488.36) | 0.20  (0.05 to 0.36) | 236.30  (57.04 to 435.09) | 0.20  (0.05 to 0.36) | 239.45 (60.46 to 436.23) |
| Angola | 76.45  (33.60 to 120.81) | 740.89  (325.66 to 1,170.82) | 123.79  (57.81 to 194.01) | 836.86  (390.83 to 1,311.52) | 121.77  (46.18 to 201.80) | 558.07  (211.63 to 924.87) | 134.33  (42.72 to 231.13) | 445.71  (141.75 to 766.90) |
| Antigua and Barbuda | 0.21  (0.08 to 0.36) | 351.25 (123.79 to 588.60) | 0.23  (0.07 to 0.39) | 298.42  (94.69 to 508.89) | 0.22  (0.07 to  0.38) | 259.89  (80.75 to  445.97) | 0.21  (0.06 to  0.37) | 242.36  (72.31 to  418.17) |
| Argentina | 270.92 (139.20 to 401.35) | 817.98 (420.29 to 1211.78) | 238.25 (110.97 to 367.22) | 647.29 (301.48 to 997.69) | 249.07 (107.44 to 391.27) | 605.56 (261.23 to 951.27) | 248.82 (102.05 to 394.25) | 551.52 (226.19 to 873.87) |
| Armenia | 34.10 (17.55 to 50.69) | 998.62 (513.84 to 1484.55) | 43.24 (24.69 to 61.96) | 1302.66 (743.72 to 1866.56) | 24.79 (12.49 to 37.60) | 798.28 (401.96 to 1210.50) | 19.45  (8.92 to 29.96) | 644.14 (295.27 to 992.11) |
| Australia | 45.76 (13.17 to 79.50) | 271.39  (78.11 to 471.55) | 44.74 (11.59 to 78.62) | 236.91  (61.39 to 416.27) | 50.25 (13.83 to 88.05) | 232.70  (64.04 to 407.70) | 72.91 (23.45 to 124.88) | 296.76 (95.44 to 508.28) |
| Austria | 34.00 (11.73 to 56.07) | 437.68 (150.98 to 721.80) | 29.86  (9.76 to 50.72) | 372.27 (121.66 to 632.27) | 27.06  (8.29 to 47.25) | 323.17  (99.04 to 564.32) | 26.72  (7.58 to 46.92) | 299.72 (85.00 to 526.27) |
| Azerbaijan | 65.72 (36.21 to 95.80) | 896.52 (493.89 to 1306.72) | 119.57 (74.54 to 166.19) | 1447.23 (902.26 to 2011.52) | 73.71 (39.49 to 108.38) | 794.04 (425.42 to 1167.55) | 61.37 (27.19 to 94.87) | 597.08 (264.49 to 922.99) |
| Bahamas | 0.62  (0.17 to 1.09) | 242.00  (66.01 to 426.64) | 0.72  (0.20 to 1.26) | 232.83  (63.62 to 406.35) | 0.79  (0.21 to 1.38) | 222.94  (59.64 to 389.24) | 0.85  (0.24 to 1.48) | 224.25 (63.68 to 393.28) |
| Baharain | 5.70  (3.33 to 8.12) | 1121.85 (654.89 to 1598.36) | 6.44  (3.58 to 9.36) | 992.15 (551.21 to 1441.90) | 10.81  (5.36 to 16.14) | 865.81 (429.36 to 1292.72) | 10.85  (4.88 to 16.81) | 752.41 (338.35 to 1164.96) |
| Bangladesh | 3068.33 (1954.60 to 4194.50) | 2813.58 (1792.32 to 3846.26) | 2616.93 (1626.63 to 3644.09) | 2045.86 (1271.67 to 2848.87) | 1597.99 (831.16 to 2342.09) | 1105.08 (574.78 to 1619.66) | 869.55 (343.20 to 1394.83) | 545.99 (215.50 to 875.82) |
| Barbados | 0.83  (0.28 to 1.40) | 325.46 (108.56 to 552.90) | 0.75  (0.24 to 1.28) | 290.69  (93.04 to 498.62) | 0.78  (0.24 to 1.34) | 277.47  (84.88 to 476.09) | 0.81  (0.26 to 1.39) | 273.44 (86.19 to 468.13) |
| Belarus | 95.13 (51.15 to 139.35) | 908.57 (488.52 to 1330.85) | 108.39 (62.60 to 156.03) | 1059.01 (611.62 to 1524.44) | 69.25 (33.95 to 103.81) | 715.53 (350.78 to 1072.69) | 52.40 (22.35 to 82.21) | 551.54 (235.25 to 865.34) |
| Belgium | 45.06 (16.97 to 74.13) | 451.48 (170.03 to 742.79) | 39.54 (12.88 to 66.66) | 385.46 (125.51 to 649.82) | 37.10 (11.52 to 63.84) | 341.09 (105.88 to 586.94) | 36.57 (10.74 to 63.20) | 320.26 (94.04 to 553.45) |
| Belize | 1.41  (0.69 to 2.11) | 756.73 (373.39 to 1137.34) | 1.42  (0.62 to 2.22) | 593.75 (256.97 to 926.94) | 1.70  (0.69 to 2.67) | 516.12 (210.12 to 813.22) | 1.96  (0.77 to 3.13) | 476.74 (188.38 to 764.25) |
| Benin | 55.39  (29.37 to  82.56) | 1,141.43  (605.15 to  1,701.43) | 75.56  (39.13 to  111.68) | 1,126.49  (583.42 to  1,664.95) | 98.03  (51.99 to  144.72) | 1,044.03  (553.68 to  1,541.29) | 113.88  (52.90 to  174.49) | 899.15  (417.63 to  1,377.69) |
| Bermuda | 0.11  (0.03 to 0.20) | 190.85  (45.57 to 341.48) | 0.11  (0.03 to 0.20) | 174.33  (41.49 to 312.26) | 0.09  (0.02 to 0.17) | 141.25  (30.12 to 257.57) | 0.08  (0.02 to 0.16) | 131.87 (28.27 to 246.46) |
| Bhutan | 17.57 (11.80 to 23.59) | 2870.93 (1928.17 to 3854.14) | 14.80  (9.69 to 20.26) | 2282.05 (1495.06 to 3125.24) | 12.96  (8.23 to 17.85) | 1771.28 (1124.60 to 2439.88) | 10.68  (6.54 to 15.02) | 1415.77 (866.63 to 1991.40) |
| Bolivia  (Plurinational State of) | 66.81  (39.21 to 95.12) | 1040.45 (610.70 to 1481.33) | 76.98  (44.59 to 110.23) | 921.99 (534.03 to 1320.18) | 80.89  (43.83 to 118.13) | 795.22 (430.92 to 1161.37) | 74.37  (36.04 to 113.78) | 619.18 (300.03 to 947.26) |
| Bosnia and Herzegovina | 64.00 (38.13 to 90.15) | 1410.34 (840.25 to 1986.63) | 44.44 (25.65 to 63.63) | 1085.26 (626.30 to 1553.94) | 27.72 (14.17 to 41.78) | 734.92 (375.72 to 1107.58) | 19.40  (9.02 to 30.21) | 587.76 (273.45 to 915.38) |
| Botswana | 6.28  (2.09 to  10.53) | 482.46  (160.34 to  808.92) | 6.60  (2.13 to 511.08) | 391.91  (126.35 to  658.07) | 6.59  (1.97 to  11.43) | 329.78  (98.66 to  572.25) | 6.85  (1.91 to 11.92) | 292.77  (81.79 to  509.48) |
| Brazil | 775.41 (334.90 to 1227.34) | 520.98 (225.01 to 824.63) | 836.35 (355.48 to 1338.91) | 479.99 (204.02 to 768.42) | 784.77 (296.34 to 1297.24) | 395.69 (149.42 to 654.08) | 749.62 (261.53 to 1252.50) | 345.98 (120.71 to 578.08) |
| Brunei Darussalam | 0.44  (0.05 to 0.92) | 169.93  (17.88 to 354.22) | 0.63  (0.08 to 1.26) | 188.29  (23.05 to 377.85) | 0.50  (0.04 to 1.10) | 127.83  (9.43 to 278.70) | 0.43  (0.02 to 1.03) | 98.45  (4.96 to 234.51) |
| Bulgaria | 63.44 (33.86 to 92.52) | 730.76 (390.08 to 1065.80) | 62.97 (35.44 to 92.37) | 792.60 (446.09 to 1162.74) | 42.93 (21.53 to 65.13) | 577.05 (289.42 to 875.49) | 31.57 (14.15 to 49.59) | 455.23 (203.98 to 715.10) |
| Burkina Faso | 128.56  (68.34 to  194.33) | 1,345.07  (715.02 to  2,033.26) | 159.59  (80.94 to  240.98) | 1.293.95  (656.26 to  1,953.79) | 212.93  (115.09 to  314.49) | 1,255.55  (678.59 to  1,854.35) | 230.35  (110.19 to  349.02) | 1,015.13  (485.59 to  1,538.10) |
| Burundi | 85.86  (49.44 to  124.12) | 1,541.22  (887.42 to  2,228.08) | 111.92  (66.12 to  156.79) | 1,774.54  (1,048.29 to  2,485.98) | 167.18  (102.97 to  235.14) | 1,863.27  (1,147.61 to  2,620.65) | 209.22  (124.64 to  297.72) | 1,753.06  (1,044.37 to  2,494.61) |
| Cabo Verde | 2.62  (1.11 to  4.21) | 744.19  (316.28 to  1,196.40) | 2.69  (1.03 to  4.35) | 598.60  (229.12 to  968.26) | 2.25  (0.75 to  3.80) | 439.41  (146.13 to  741.85) | 2.07  (0.60 to  3.60) | 366.61  (105.87 to  638.29) |
| Cambodia | 319.85 (216.15 to 427.01) | 3082.83 (2083.26 to 4115.62) | 356.85 (239.93 to 478.25) | 2832.20 (1904.23 to 3795.70) | 293.23 (193.24 to 396.91) | 2016.97 (1329.19 to 2730.12) | 235.93 (145.32 to 333.32) | 1420.98 (875.24 to 2007.57) |
| Cameroon | 73.41  (30.33 to  116.08) | 706.46  (291.93 to  1,117.12) | 129.26  (62.48 to  197.07) | 860.30  (415.85 to  1,311.58) | 177.45  (81.76 to  271.73) | 807.14  (371.92 to  1,236.02) | 198.96  (81.24 to  324.52) | 683.65  (279.15 to  1,115.13) |
| Canada | 84.96 (35.33 to 146.58) | 311.71 (129.64 to 537.77) | 90.42 (37.96 to 155.77) | 298.39 (125.28 to 514.05) | 165.77 (60.81 to 274.21) | 493.63 (181.08 to 816.57) | 168.16 (58.00 to 282.11) | 460.45 (158.83 to 772.48) |
| Central African Republic | 37,415.73  (19,824.68 to 55,017.64) | 1,363.52  (722.46 to 2,004.98) | 53.18  (28.73 to 77.87) | 1,467.36  (792.84 to  2,148.57) | 70.29  (39.01 to 101.14) | 1,522.52  (845.08 to 2,190.89) | 89.86  (52.07 to 129.03) | 1,695.49  (982.46 to 2,434.62) |
| Chad | 83.22  (47.83 to  120.43) | 1,382.00  (794.33 to  1,999.82) | 115.09  (64.20 to  168.10) | 1,391.38  (776.18 to  2,032.29) | 132.60  (70.72 to  195.75) | 1,126.36  (600.74 to  1,662.85) | 164.56  (82.09 to  248.90) | 1,003.51  (500.59 to  1,517.76) |
| Chile | 114.76 (58.51 to 170.92) | 864.14 (440.60 to 1287.00) | 89.33 (35.21 to 142.63) | 590.29 (232.69 to 942.52) | 80.59 (28.80 to 132.71) | 483.64 (172.85 to 796.44) | 77.23 (26.40 to 129.20) | 424.39 (145.08 to 709.97) |
| China | 8650.68 (4696.83 to 12772.47) | 730.83 (396.80 to 1079.05) | 7896.98 (4191.05 to 11845.03) | 607.25 (322.28 to 910.84) | 5996.20 (2682.29 to 9394.81) | 437.23 (196.57 to 688.50) | 4930.87 (1897.71 to 7980.06) | 346.67 (133.42 to 561.05) |
| Colombia | 194.25 (93.72 to 300.66) | 596.83 (287.93 to 923.76) | 197.74 (85.08 to 311.64) | 495.15 (213.05 to 780.34) | 183.80 (71.42 to 300.35) | 411.13 (159.76 to 671.83) | 153.72 (51.03 to 260.59) | 321.74 (106.82 to 545.44) |
| Comoros | 4.30  (2.00 to  6.75) | 924.11  (429.74 to  1,448.44) | 5.43  (2.49 to  8.44) | 980.70  (450.21 to  1,523.93) | 6.04  (2.86 to  9.28) | 944.53  (447.84 to  1,452.34) | 6.06  (2.50 to  9.78) | 848.19  (349.47 to  1,369.63) |
| Congo | 13.21  (4.61 to 22.03) | 540.41  (188.44 to  901.16) | 18.81  (6.92 to  30.89) | 590.89  (217.28 to  970.40) | 22.22  (7.62 to  37.58) | 527.38  (180.83 to  891.91) | 26.15  (9.30 to  43.32) | 496.52  (176.54 to  822.69) |
| Cook Islands | 0.10  (0.05 to 0.16) | 534.57 (238.38 to 828.95) | 0.08  (0.03 to 0.13) | 423.36 (167.29 to 680.88) | 0.07  (0.03 to 0.11) | 361.92 (135.49 to 586.81) | 0.06  (0.02 to 0.09) | 308.45 (110.64 to 511.96) |
| [Costa Rica](https://www.google.com/search?rlz=1C1SQJL_enSG848SG848&sxsrf=AOaemvKVzD4xx8QgHJaXFWcgVaLL3GFlxQ:1633946229259&q=Costa+Rica&spell=1&sa=X&ved=2ahUKEwiOq86EjMLzAhVLuksFHeFrB-QQBSgAegQIARBW) | 16.38  (7.61 to 25.99) | 538.85 (250.37 to 854.91) | 18.12  (7.96 to 28.66) | 464.46 (203.99 to 734.70) | 17.97  (7.58 to 28.68) | 409.55 (172.61 to 653.52) | 15.92  (5.91 to 26.22) | 337.42 (125.25 to 555.99) |
| Côte d'Ivoire | 78.32  (30.15 to  128.17) | 640.62  (246.63 to  1,048.34) | 123.77  (49.33 to  201.59) | 721.85  (287.74 to  1,175.73) | 162.78  (68.04 to  259.28) | 751.79  (314.26 to  1,197.52) | 156.42  (54.11 to  260.52) | 597.68  (206.76 to  995.43) |
| Croatia | 25.31 (11.33 to 39.43) | 516.43 (231.18 to 804.62) | 22.47  (8.99 to 36.03) | 491.84 (196.77 to 788.61) | 17.33  (6.68 to 28.02) | 396.31 (152.70 to 640.72) | 15.71  (5.84 to 25.86) | 369.90 (137.51 to 608.84) |
| Cuba | 52.75 (21.51 to 84.56) | 487.00 (198.55 to 780.66) | 75.07 (37.73 to 114.56) | 658.95 (331.20 to 1005.60) | 61.59 (28.79 to 94.69) | 537.18 (251.11 to 825.87) | 48.84 (21.00 to 78.54) | 430.00 (184.85 to 691.46) |
| Cyprus | 4.43  (1.77 to 7.15) | 569.24 (227.92 to 919.16) | 4.29  (1.51 to 7.13) | 467.83 (164.42 to 776.69) | 4.36  (1.40 to 7.40) | 389.15 (124.91 to 659.45) | 5.18  (1.68 to 8.76) | 394.45 (127.92 to 667.05) |
| Czechia | 52.29 (24.37 to 80.36) | 507.81 (236.64 to 780.43) | 44.89 (18.25 to 71.82) | 438.26 (178.20 to 701.22) | 36.15 (12.91 to 60.00) | 345.20 (123.28 to 572.94) | 33.05 (11.54 to 54.55) | 310.50 (108.39 to 512.51) |
| Democratic People’s Republic of Korea | 227.07 (141.81 to 311.24) | 1078.47 (673.51 to 1478.25) | 326.13 (211.62 to 448.97) | 1364.13 (885.17 to 1877.98) | 358.29 (233.33 to 488.16) | 1404.61 (917.30 to 1919.11) | 383.64 (251.07 to 520.21) | 1462.44 (957.07 to 1983.06) |
| Democratic Republic of Congo | 454.41  (238.69 to  668.22) | 1,177.51  (1,731.55 to  618.51) | 924.36  (1,298.21 to  547.00) | 1,807.49  (2,538.50 to  1,069.59) | 1,372.19  (1,925.18 to  845.55) | 2,003.42  (2,810.79 to  1,234.51) | 1,326.84  (762.03 to  1,925.42) | 1,513.44  (869.20 to  2,196.21) |
| Denmark | 5.74  (0.84 to 12.03) | 111.64  (16.33 to 233.91) | 5.15  (0.72 to 11.21) | 96.46  (13.58 to 209.98) | 4.52  (0.62 to 9.95) | 81.68  (11.20 to 179.81) | 4.06  (0.47 to 9.45) | 69.91  (8.14 to 162.79) |
| Djibouti | 3.42  (1.44 to  5.43) | 703.92  (296.89 to  1,118.21) | 5.30  (2.37 to  8.29) | 819.70  (365.67 to  1,280.77) | 6.97  (3.04 to  11.06) | 757.02  (329.72 to  1,200.28) | 7.17  (2.41 to  11.97) | 595.91  (200.27 to  995.50) |
| Dominica | 0.41  (0.17 to 0.65) | 555.23 (234.50 to 874.21) | 0.32  (0.13 to 0.52) | 452.11 (180.90 to 736.98) | 0.27  (0.10 to 0.45) | 393.62 (141.97 to 652.46) | 0.25  (0.08 to 0.42) | 366.59 (123.54 to 617.17) |
| Dominican Republic | 43.58 (18.32 to 67.94) | 605.02 (254.38 to 943.17) | 41.76 (15.54 to 68.74) | 481.98 (179.31 to 793.28) | 38.07 (12.77 to 64.31) | 387.80 (130.05 to 655.14) | 34.40 (10.95 to 58.98) | 316.10 (100.64 to 541.97) |
| Ecuador | 62.38  (29.88 to 94.03) | 622.21 (298.06 to 937.84) | 68.69 (32.28 to 106.05) | 557.18 (261.85 to 860.28) | 73.06 (31.28 to 113.70) | 489.04 (209.37 to 761.13) | 72.79 (27.31 to 117.27) | 413.84 (155.26 to 666.75) |
| Egypt | 1763.66 (1163.15 to 2368.06) | 3166.47 (2088.31 to 4251.61) | 1856.22 (1192.30 to 2526.16) | 2742.02 (1761.28 to 3731.68) | 1932.77 (1221.70 to 2646.09) | 2307.08 (1458.30 to 3158.55) | 1976.07 (1184.54 to 2773.71) | 1994.63 (1195.67 to 2799.76) |
| El Salvador | 44.86 (23.43 to 66.44) | 851.67 (444.85 to 1261.25) | 39.52 (19.38 to 60.19) | 680.33 (333.68 to 1036.13) | 35.22 (16.75 to 54.85) | 586.31 (278.81 to 913.14) | 29.27 (11.37 to 48.28) | 467.86 (181.70 to 771.80) |
| England | 201.21 (63.93 to 347.07) | 420.15 (133.49 to 724.73) | 181.11 (53.78 to 320.30) | 364.01 (108.08 to 643.77) | 170.02 (48.87 to 308.68) | 318.98  (91.69 to 579.11) | 169.95 (46.51 to 312.42) | 300.39 (82.21 to 552.19) |
| Equatorial Guinea | 5.16  (2.53 to  7.92) | 1,199.80  (587.62 to  1,841.25) | 3.61  (1.34 to  6.08) | 540.93  (200.51 to  909.92) | 2.04  (0.38 to  3.88) | 195.67  (36.52 to  372.16) | 2.86  (0.52 to  5.34) | 201.34  (36.31 to  376.09) |
| Eritrea | 50.22  (28.60 to  72.45) | 1,673.26  (952.83  2,413.75) | 45.70  (22.30 to  68.98) | 1,115.07  (544.13 to  1,683.23) | 66.76  (34.27 to  100.54) | 1,181.01  (606.22 to  1,778.55) | 70,113.25  (33,751.90 to  108,095.08) | 1,044.72  (502.92 to  1,610.66) |
| Estonia | 9.42  (4.60 to 14.52) | 600.48 (293.50 to 925.79) | 8.12  (3.92 to 12.67) | 582.82 (281.06 to 908.91) | 5.26  (2.00 to 8.52) | 394.55 (150.18 to 639.25) | 4.48  (1.61 to 7.48) | 341.37 (123.00 to 569.63) |
| Ethiopia | 1,010.91  (596.47 to  1,443.56) | 1,967.09  (2,808.96 to  1,160.64) | 1,469.26  (879.54 to  2,087.66) | 2,159.66  (1,292.83 to  3,068.65) | 1,537.00  (901.09 to  2,192.73) | 1,790.08  (1,049.46 to  2,553.78) | 1,261.75  (666.40 to  1,885.20) | 1,172.73  (619.38 to  1,752.19) |
| Eswatini | 4.85  (1.90 to  7.90) | 601.05  (235.55 to  978.99) | 5.06  (1.78 to  8.36) | 497.98  (175.63 to  823.25) | 4.92  (1.79 to  8.19) | 457.72  (166.51 to  761.30) | 4.85  (1.67 to  8.05) | 424.41  (146.59 to  704.91) |
| Fiji | 7.40  (4.33 to 10.69) | 974.64 (570.64 to 1407.68) | 7.08  (4.01 to 10.24) | 867.95 (490.96 to 1255.16) | 7.07  (3.84 to 10.33) | 807.83 (438.98 to 1181.04) | 6.66  (3.37 to 9.95) | 730.40 (369.42 to 1092.28) |
| Finland | 28.06 (11.45 to 45.10) | 560.04 (228.60 to 900.27) | 25.45  (9.77 to 41.59) | 490.41 (188.20 to 801.45) | 22.65  (8.06 to 38.27) | 420.80 (149.70 to 711.01) | 22.11  (7.45 to 37.49) | 399.60 (134.62 to 677.35) |
| France | 346.14 (147.66 to 531.50) | 599.18 (255.60 to 920.03) | 336.52 (137.66 to 525.61) | 561.39 (229.64 to 876.82) | 303.62 (114.93 to 471.53) | 475.89 (180.13 to 739.07) | 280.53 (100.74 to 446.74) | 423.74 (152.16 to 674.79) |
| Gabon | 2.530  (0.52 to  4.68) | 255.16  (52.49 to  471.83) | 3.21  (0.69 to  5.89) | 257.67  (55.44 to  471.85) | 4.08  (0.98 to  7.39) | 271.02  (64.88 to  491.21) | 4.50  (1.05 to  8.17) | 256.87  (60.13 to  466.69) |
| Gambia | 9.18  (3.98 to  14.26) | 925.12  (400.84 to  1,437.58) | 13.27  (6.11 to  20.79) | 975.75  (448.95 to  1,528.64) | 16.74  (7.64 to  25.99) | 940.43  (429.47 to  1,460.30) | 20.73  (9.21 to  32.09) | 923.08  (410.22 to  1,428.78) |
| Georgia | 38.84 (18.95 to 57.91) | 705.04 (343.92 to 1051.26) | 59.10 (35.19 to 83.10) | 1249.61 (744.03 to 1757.01) | 33.80 (17.67 to 49.79) | 848.70 (443.76 to 1250.07) | 23.06 (10.05 to 35.58) | 629.36 (274.37 to 971.00) |
| Germany | 309.98 (86.97 to 528.73) | 387.76 (108.80 to 661.40) | 283.87 (77.19 to 490.09) | 344.86  (93.78 to 595.37) | 235.92 (59.30 to 417.05) | 288.91  (72.61 to 510.72) | 215.41 (48.57 to 387.66) | 253.68 (57.19 to 456.53) |
| Ghana | 150.47  (71.78 to  230.58) | 1,002.01  (478.03 to  1,535.53) | 171.65  (84.64 to  263.50) | 894.88  (441.26 to  1,373.74) | 207.11  (99.11 to  314.63) | 818.77  (391.81 to  1,243.85) | 189.88  (76.91 to  313.03) | 602.10  (243.89 to  992.60) |
| Greece | 59.51 (25.28 to 94.43) | 572.82 (243.34 to 908.89) | 53.50 (19.94 to 87.28) | 482.34 (179.78 to 786.94) | 41.89 (13.43 to 71.38) | 378.16 (121.21 to 644.35) | 41.72 (14.05 to 70.98) | 403.58 (135.91 to 686.60) |
| Greenland | 0.19  (0.06 to 0.34) | 350.72 (114.76 to 609.92) | 0.20  (0.06 to 0.34) | 356.28 (114.83 to 607.40) | 0.25  (0.09 to 0.41) | 437.16 (151.74 to 730.14) | 0.22  (0.07 to 0.37) | 387.96 (131.00 to 657.84) |
| Grenada | 0.53  (0.25 to 0.82) | 622.20 (286.80 to 955.27) | 0.50  (0.22 to 0.81) | 491.37 (217.19 to 788.29) | 0.41  (0.15 to 0.67) | 382.61 (138.00 to 626.44) | 0.34  (0.11 to 0.57) | 329.38 (106.60 to 551.62) |
| Guam | 0.35  (0.11 to 0.59) | 255.77  (78.35 to 433.69) | 0.42  (0.13 to 0.43) | 262.32  (84.75 to 446.17) | 0.40  (0.13 to 0.68) | 243.24  (76.79 to 414.64) | 0.40  (0.12 to 0.68) | 232.44 (70.34 to 396.81) |
| Guatemala | 60.43 (28.71 to 93.69) | 758.49 (360.43 to 1176.00) | 75.74 (35.85 to 116.61) | 694.58 (328.79 to 1069.32) | 87.78 (40.25 to 136.80) | 603.86 (276.86 to 941.08) | 90.26 (37.03 to 147.84) | 507.74 (208.34 to 831.66) |
| Guinea | 64.48  (30.22 to  98.72) | 1,042.32  (488.56 to  1,595.69) | 86.81  (42.43 to  129.52) | 1,087.13  (531.32 to  1,621.86) | 120.68  (55.34 to  185.73) | 954.53  (437.72 to  1,469.02) | 113.05  (59.10 to  169.81) | 1,133.75  (592.73 to  1,702.98) |
| Guinea-Bissau | 11.10  (5.45 to 16.89) | 1,102.33  (541.34 to  1,676.93) | 13.47  (6.08 to  20.85) | 1,077.76  (486.80 to  1,667.98) | 17.93  (9.28 to  27.06) | 1,150.95  (595.93 to  1,737.20) | 18.57  (8.31 to  29.32) | 976.96  (436.87 to  1,542.18) |
| Guyana | 6.66  (3.18 to 10.20) | 865.45 (413.27 to 1324.62) | 5.33  (2.51 to 8.42) | 693.86 (326.30 to 1095.54) | 4.52  (1.95 to 7.15) | 604.56 (261.51 to 956.38) | 3.68  (1.31 to 6.00) | 477.43 (170.51 to 777.95) |
| Haiti | 76.11 (40.34 to 113.65) | 1197.50 (634.68 to 1788.21) | 104.94 (57.91 to 154.45) | 1280.09 (706.44 to 1884.10) | 137.45 (79.23 to 197.26) | 1330.93 (767.17 to 1910.10) | 151.17 (82.03 to 220.61) | 1218.94 (661.45 to 1778.78) |
| Honduras | 43.78 (22.87 to 65.04) | 929.86 (485.77 to 1381.29) | 54.90 (29.88 to 81.64) | 889.65 (484.23 to 1323.03) | 64.95 (33.55 to 96.83) | 813.24 (419.99 to 1212.33) | 66.79 (30.60 to 102.51) | 680.52 (311.81 to 1044.45) |
| Hungary | 56.41 (26.02 to 86.99) | 542.80 (250.40 to 837.06) | 52.92 (22.58 to 83.74) | 518.72 (221.30 to 820.81) | 39.93 (15.70 to 64.55) | 400.67 (157.50 to 647.77) | 33.87 (12.79 to 55.23) | 350.05 (132.17 to 570.84) |
| Iceland | 1.07  (0.35 to 1.85) | 423.02 (136.77 to 729.27) | 1.11  (0.35 to 1.90) | 396.33 (126.50 to 681.50) | 1.05  (0.30 to 1.87) | 330.86  (0.09 to 0.59) | 1.05  (0.27 to 1.86) | 304.49 (79.32 to 540.35) |
| India | 43577.84 (30215.37 to 57424.52) | 5093.33 (3531.54 to 6711.72) | 50446.22 (35147.25 to 66437.61) | 4888.22 (3405.76 to 6437.78) | 56815.99 (39335.49 to 74763.95) | 4614.10 (3196.11 to 6071.67) | 58532.66 (40555.71 to 77111.80) | 4208.84 (2916.19 to 5544.79) |
| Indonesia | 3448.45 (2265.45 to 4687.92) | 1860.23 (1222.07 to 2528.85) | 3143.81 (1992.72 to 4317.41) | 1471.64 (932.81 to 2021.01) | 2998.37 (1881.07 to 4158.48) | 1245.64 (781.47 to 1727.60) | 2347.73 (1335.71 to 3372.79) | 904.83 (514.79 to 1299.90) |
| Iran | 1418.15 (946.71 to 1900.35) | 2422.51 (1617.19 to 3246.23) | 1406.57 (920.29 to 1911.94) | 2089.63 (1367.20 to 2840.42) | 1206.61 (762.69 to 1666.94) | 1575.61 (995.93 to 2176.72) | 1169.06 (699.22 to 1662.27) | 1386.83 (829.46 to 1971.90) |
| Iraq | 326.26 (199.98 to 451.25) | 1854.10 (1136.49 to 2564.40) | 655.67 (419.02 to 893.17) | 2436.08 (1556.84 to 3318.51) | 686.66 (431.22 to 947.65) | 2019.83 (1268.45 to 2787.56) | 672.80 (400.99 to 961.53) | 1597.37 (952.02 to 2282.87) |
| Ireland | 22.97  (9.37 to 36.84) | 637.71 (260.27 to 1023.09) | 17.92  (6.16 to 29.85) | 463.48 (159.39 to 771.79) | 16.07  (4.87 to 28.10) | 349.34 (105.88 to 610.81) | 14.18  (3.53 to 25.79) | 288.71 (71.91 to 525.27) |
| Israel | 28.00 (11.03 to 45.36) | 564.40 (222.39 to 914.21) | 30.49 (11.12 to 51.20) | 476.96 (174.00 to 800.88) | 34.36 (11.95 to 57.34) | 437.77 (152.31 to 730.62) | 36.77 (11.74 to 62.04) | 395.01 (126.12 to 666.36) |
| Italy | 261.08 (98.06 to 426.12) | 459.67 (172.64 to 750.24) | 227.49 (80.79 to 377.49) | 401.48 (142.58 to 666.19) | 224.50 (79.65 to 376.25) | 372.13 (132.04 to 623.68) | 222.13 (77.88 to 368.44) | 368.29 (129.13 to 610.87) |
| Jamaica | 12.87  (5.16 to 20.69) | 544.48 (218.42 to 875.13) | 12.52  (5.18 to 20.30) | 477.53 (197.70 to 774.52) | 12.29  (4.62 to 20.26) | 446.12 (167.74 to 735.54) | 11.97  (4.26 to 19.73) | 426.68 (151.61 to 701.93) |
| Japan | 435.77 (119.38 to 763.43) | 346.21  (94.84 to 606.54) | 399.57 (104.80 to 707.35) | 309.67  (81.22 to 548.20) | 314.88 (63.58 to 581.69) | 242.12  (48.89 to 447.29) | 252.85 (42.82 to 482.10) | 197.86 (33.50 to 377.26) |
| Jordan | 84.35 (54.61 to 115.61) | 2235.42 (1447.23 to 3063.97) | 108.38 (71.50 to 148.37) | 2291.77 (1511.95 to 3137.33) | 135.90 (82.51 to 188.22) | 1866.56 (1133.27 to 2585.20) | 193.93 (115.96 to 275.67) | 1666.55 (996.46 to 2369.00) |
| Kazakhstan | 120.31 (59.92 to 182.56) | 735.03 (366.09 to 1115.35) | 138.21 (77.46 to 203.23) | 921.25 (516.33 to 1354.65) | 100.61 (50.85 to 153.74) | 620.86 (313.79 to 948.74) | 87.13 (35.81 to 138.57) | 473.73 (194.69 to 753.42) |
| Kenya | 224.03  (113.30 to  337.46) | 965.93  (488.50 to  1,454.97) | 311.00  (159.33 to  466.11) | 1,001.04  (512.85 to  1,500.31) | 395.81  (205.33 to  595.56) | 972.94  (504.72 to  1,463.93) | 397.69  (186.72 to  618.13) | 791.77  (371.74 to  1,230.66) |
| Kiribati | 1.19  (0.76 to 1.63) | 1609.69 (1029.55 to 2197.60) | 1.50  (0.95 to 2.04) | 1718.77 (1091.01 to 2341.05) | 1.84  (1.18 to 2.52) | 1744.01 (1114.86 to 2386.61) | 2.02  (1.31 to 2.77) | 1704.41 (1103.27 to 2335.22) |
| Kuwait | 15.13  (7.85 to 22.82) | 859.74 (446.42 to 1297.04) | 13.10  (6.01 to 20.44) | 684.71 (314.24 to 1068.24) | 17.49  (7.40 to 28.07) | 585.68 (247.75 to 939.71) | 26.37 (11.33 to 41.67) | 595.74 (255.93 to 941.34) |
| Kyrgyzstan | 61.75 (38.20 to 85.88) | 1383.88 (856.11 to 1924.72) | 96.03 (63.18 to 131.03) | 1910.84 (1257.20 to 2607.32) | 97.54 (62.23 to 133.51) | 1735.18 (1107.05 to 2375.02) | 96.48 (60.01 to 134.57) | 1476.24 (918.16 to 2059.11) |
| Lao People’s Democratic Republic | 112.49 (76.41 to 150.43) | 2709.56 (1840.45 to 3623.32) | 123.01 (82.37 to 164.27) | 2296.09 (1537.49 to 3066.03) | 109.12 (71.42 to 148.25) | 1717.12 (1123.88 to 2332.79) | 80.80 (48.08 to 115.35) | 1128.84 (671.64 to 1611.37) |
| Latvia | 15.56  (7.06 to 23.85) | 585.21 (265.70 to 896.93) | 17.23  (9.17 to 25.65) | 722.41 (384.57 to 1075.86) | 9.89  (4.26 to 15.50) | 467.65 (201.36 to 732.81) | 7.17  (2.64 to 11.65) | 374.42 (138.06 to 608.38) |
| Lebanon | 63.95  (41.97 to 86.43) | 1952.51 (1281.48 to 2639.04) | 66.01 (42.48 to 91.47) | 1782.83 (1147.26 to 2470.50) | 60.83 (37.34 to 85.42) | 1467.11 (900.51 to 2060.33) | 70.30 (41.46 to 99.80) | 1357.99 (800.91 to 1927.71) |
| Lesotho | 23.55  (13.15 to  34.24) | 1.30  (0.73 to  1.89) | 22.18  (11.35 to  32.75) | 1,121.62  (574.24 to  1,656.20) | 18.29  (9.04 to  27.97) | 929.70  (459.39 to  1,422.17) | 15.51  (6.77 to  24.53) | 741.50  (323.73 to  1,172.68) |
| Liberia | 23.09  (11.72 to  34.15) | 1,175.33  (596.60 to  1,738.64) | 59.98  (36.80 to  84.83) | 2,049.97  (1,257.64 to  2,899.22) | 66.37  (38.14 to  94.76) | 1,635.56  (939.95 to  2,335.32) | 64.97  (35.01 to  95.03) | 1,356.48  (730.92 to  1,984.03) |
| Libya | 56.39  (33.62 to 78.67) | 1330.87 (793.44 to 1856.88) | 70.41 (42.55 to 99.53) | 1385.98 (837.58 to 1959.17) | 72.37 (40.69 to 105.23) | 1189.67 (668.89 to 1729.88) | 110.81 (67.72 to 156.14) | 1645.17 (1005.45 to 2318.09) |
| Lithuania | 22.28 (10.76 to 33.83) | 606.41 (292.96 to 920.81) | 24.87 (13.19 to 36.83) | 706.51 (374.54 to 1046.13) | 13.75  (5.69 to 21.76) | 445.04 (184.24 to 704.27) | 9.53  (3.33 to 15.90) | 340.98 (119.30 to 568.93) |
| Luxembourg | 1.31  (0.39 to 2.26) | 342.86 (101.77 to 592.76) | 1.20  (0.33 to 2.12) | 276.59  (75.60 to 489.41) | 1.17  (0.29 to 2.16) | 232.45  (58.27 to 429.09) | 1.34  (0.33 to 2.49) | 216.87 (52.81 to 401.91) |
| Madagascar | 132.65  (65.35 to  199.97) | 1,109.88  (546.81 to  1,673.21) | 191.11  (102.40 to  287.23) | 1,206.75  (646.64 to  1,813.73) | 253.59  (132.87 to  381.21) | 1,200.76  (629.12 to  1,805.03) | 286.04  (143.24 to  437.35) | 1,071.68  (1,638.59 to  536.67) |
| Malawi | 152.75  (87.37 to  220.78) | 1,598.48  (914.37 to  2,310.44) | 175.16  (98.54 to  255.20) | 1,579.19  (888.40 to  2,300.83) | 217.77  (120.62 to  316.77) | 1,524.62  (844.47 to  2,217.64) | 249.03  (129.56 to  364.90) | 1,350.33  (702.50 to  1,978.60) |
| Malaysia | 174.56 (96.12 to 256.73) | 988.72 (544.43 to 1454.12) | 172.57 (89.38 to 260.61) | 724.45 (375.24 to 1094.05) | 162.56 (76.26 to 253.01) | 577.66 (270.99 to 899.11) | 132.98 (56.50 to 217.29) | 424.83 (180.49 to 694.17) |
| Maldives | 3.37 (2.09 to 4.67) | 1518.58 (940.01 to 2105.99) | 3.20  (1.94 to 4.54) | 1132.02 (686.95 to 1604.53) | 2.76  (1.48 to 4.11) | 769.20 (413.07 to 1145.65) | 2.79  (1.25 to 4.35) | 560.41 (250.12 to 873.36) |
| Mali | 110.40  (56.06 to  166.60) | 1,272.95  (646.37 to  1,920.98) | 128.03  (64.52 to  194.22) | 1,153.78  (581.41 to  1,750.30) | 169.49  (85.74 to  255.31) | 1,066.23  (539.36 to  1,606.16) | 212.47  (99.41 to  327.41) | 969.39  (453.56 to  1,493.83) |
| Malta | 2.31  (0.91 to 3.71) | 622.64 (245.06 to 999.63) | 1.90  (0.64 to 3.28) | 472.38 (159.84 to 816.01) | 1.68  (0.51 to 2.88) | 395.49 (120.94 to 679.56) | 1.46  (0.40 to 2.57) | 331.43 (90.79 to 584.75) |
| Marshall Islands | 0.69  (0.42 to 0.95) | 1499.55 (914.14 to 2072.44) | 0.77  (0.49 to 1.06) | 1469.54 (931.83 to 2038.30) | 0.75 (0.47 to 1.03) | 1363.73 (852.78 to 1878.18) | 0.72  (0.45 to 1.00) | 1260.88 (787.65 to 1762.36) |
| Mauritania | 16.72  (7.48 to  26.01) | 808.90  (362.06 to  1,258.47) | 20.28  (8.90 to  32.79) | 775.73  (340.33 to  1,254.55) | 22.70  (8.85 to  36.17) | 683.28  (266.34 to  1,088.94) | 24.86  (9.56 to  40.23) | 619.18  (238.03 to  1,002.11) |
| Mauritius | 11.78 (6.71 to 16.96) | 1071.06 (609.78 to 1541.86) | 9.58  (5.05 to 14.27) | 789.50 (416.04 to 1176.14) | 7.78  (3.75 to 11.78) | 612.82 (295.75 to 928.50) | 5.60  (2.21 to 9.03) | 438.73 (173.17 to 707.08) |
| Mexico | 348.18 (130.35 to 574.36) | 407.29 (152.48 to 671.86) | 371.31 (130.44 to 618.86) | 366.99 (128.92 to 611.65) | 384.88 (131.92 to 649.59) | 336.40 (115.30 to 567.78) | 372.35 (115.16 to 634.72) | 298.02 (92.17 to 508.02) |
| Micronesia (Federated States of) | 1.54  (0.96 to 2.16) | 1477.93 (921.54 to 2074.43) | 1.55  (0.96 to 2.07) | 1409.14 (873.20 to 1959.40) | 1.41 (0.89 to 1.96) | 1332.72 (840.45 to 1860.32) | 1.31  (0.80 to 1.85) | 1285.58 (782.78 to 1814.78) |
| Monaco | 0.04  (0.01 to 0.08) | 127.66  (28.20 to 255.92) | 0.04  (0.01 to 0.08) | 116.83  (24.57 to 238.63) | 0.037 (0.008 to 0.078) | 104.69  (21.23 to 219.02) | 0.033 (0.006 to 0.072) | 87.82  (16.30 to 192.16) |
| Mongolia | 29.52 (18.09 to 41.13) | 1370.64 (840.00 to 1909.76) | 35.61 (22.31 to 49.37) | 1453.58 (910.83 to 2015.27) | 30.92 (18.30 to 44.06) | 1092.15 (646.41 to 1556.28) | 25.49 (12.96 to 37.90) | 752.31 (382.70 to 1118.75) |
| Montenegro | 3.41  (1.48 to 5.33) | 544.65 (237.08 to 852.20) | 4.75  (2.45 to 7.07) | 746.76 (386.26 to 1112.51) | 3.58  (1.68 to 5.52) | 568.24 (266.72 to 875.27) | 2.95  (1.24 to 4.69) | 475.52 (199.12 to 756.56) |
| Morocco | 728.21 (488.04 to 979.76) | 2878.71 (1929.28 to 3873.11) | 775.10 (506.66 to 1050.73) | 2606.39 (1703.69 to 3533.23) | 748.91 (484.50 to 1027.93) | 2238.09 (1447.90 to 3071.91) | 670.34 (416.57 to 933.65) | 1864.54 (1158.68 to 2596.92) |
| Mozambique | 298.95  (185.16 to  417.07) | 2,287.11  (1,416.55 to  3,190.81) | 363.58  (221.78 to  513.05) | 2,073.05  (1,264.56 to 2,925.31) | 372.43  (221.46 to  525.07) | 1,619.90  (963.25 to  2,283.84) | 402.82  (228.45 to  580.21) | 1,364.18  (773.67 to  1,964.96) |
| Myanmar | 1347.78 (929.77 to 1776.54) | 3278.97 (2262.01 to 4322.08) | 1339.41 (914.98 to 1778.76) | 2886.16 (1971.59 to 3832.87) | 908.36 (604.94 to 1230.53) | 1792.84 (1193.97 to 2428.71) | 622.37 (362.41 to 884.35) | 1138.26 (662.83 to 1617.42) |
| Namibia | 6.58  (2.07 to  11.10) | 466.43  (147.06 to  787.54) | 8.52  (2.94 to  14.30) | 462.26  (159.59 to  775.60) | 8.46  (2.75 to  14.45) | 400.02  (130.27 to  683.17) | 8.37  (2.60 to  14.43) | 348.33  (108.24 to  600.51) |
| Nauru | 0.06  (0.03 to 0.09) | 572.35 (280.36 to 882.29) | 0.11  (0.06 to 0.15) | 995.22 (588.55 to 1430.37) | 0.13  (0.08 to 0.17) | 1210.56 (745.92 to 1676.42) | 0.08  (0.04 to 0.12) | 772.27 (425.97 to 1137.93) |
| Nepal | 758.59 (527.05 to 1004.72) | 3882.91 (2697.75 to 5142.75) | 834.87 (568.00 to 1112.07) | 3480.27 (2367.80 to 4635.84) | 852.03 (584.80 to 1137.73) | 3078.51 (2112.96 to 4110.80) | 792.50 (518.65 to 1069.52) | 2605.51 (1705.16 to 3516.27) |
| Netherlands | 44.83 (11.48 to 79.98) | 300.39  (76.92 to 535.96) | 39.73  (9.54 to 73.57) | 249.98  (60.05 to 462.90) | 36.13  (8.28 to 68.73) | 217.74  (49.90 to 414.18) | 34.17  (7.71 to 65.02) | 199.14 (44.93 to 379.00) |
| New Zealand | 10.14  (2.96 to 17.65) | 296.63  (86.69 to 516.37) | 9.96  (2.67 to 17.57) | 263.19  (70.48 to 464.21) | 11.34  (3.31 to 19.85) | 265.56  (77.42 to 464.91) | 14.81  (4.88 to 25.18) | 329.42 (108.58 to 560.20) |
| Niger | 93.14  (43.34 to  146.49) | 1,160.92  (540.25 to  1,825.88) | 147.75  (69.10 to  233.22) | 1,309.34  (612.41 to  2,066.82) | 234.97  (118.93 to  355.94) | 1,424.92  (721.24 to  2,158.51) | 300.89  (154.94 to  450.57) | 1,291.61  (665.11 to  1,934.15) |
| Nigeria | 705.52  (325.25 to  1,094.47) | 782.29  (360.64 to  1,213.56) | 1,113.21  (564.02 to  1,710.81) | 905.68  (458.87 to  1,391.88) | 1,088.39  (476.31 to  1,725.04) | 651.94  (285.31 to  1,033.30) | 1,200.12  (489.08 to  1,927.50) | 558.65  (227.67 to  897.25) |
| Niue | 0.02  (0.01 to 0.03) | 765.64 (398.77 to 1123.04) | 0.01  (0.01 to 0.02) | 727.19 (384.16 to 1081.43) | 0.01  (0.01 to 0.02) | 619.59 (324.86 to 931.61) | 0.01  (0.00 to 0.01) | 560.59 (270.84 to 868.04) |
| North Macedonia | 16.44  (8.94 to 23.77) | 815.78 (443.72 to 1179.35) | 17.33  (9.69 to 25.43) | 864.70 (483.65 to 1268.73) | 13.76  (6.74 to 20.95) | 650.27 (318.37 to 990.06) | 11.17  (4.62 to 17.61) | 518.91 (214.40 to 817.83) |
| Northern Ireland | 7.49  (2.39 to 12.52) | 468.96 (149.68 to 783.88) | 6.90  (2.09 to 11.76) | 404.45 (122.26 to 689.35) | 6.33  (1.73 to 10.97) | 345.90  (94.80 to 599.58) | 6.23  (1.59 to 10.82) | 321.99 (82.15 to 558.90) |
| Northern Mariana Islands | 0.12  (0.04 to 0.21) | 272.42  (89.72 to 469.41) | 0.22  (0.08 to 0.37) | 302.36 (105.76 to 506.60) | 0.18  (0.06 to 0.29) | 302.36 (105.76 to 506.60) | 0.13  (0.05 to 0.22) | 313.29 (111.02 to 525.43) |
| Norway | 12.52  (3.84 to 21.89) | 294.76  (90.36 to 515.49) | 11.72  (3.41 to 20.92) | 261.64  (76.10 to 467.04) | 11.16  (3.04 to 20.27) | 229.43  (62.54 to 416.57) | 11.52  (3.01 to 21.14) | 215.33 (56.36 to 395.15) |
| Oman | 25.34  (15.48 to 35.60) | 1304.00 (796.56 to 1832.09) | 25.53 (14.81 to 36.78) | 1110.01 (643.88 to 1598.76) | 26.07 (13.68 to 38.80) | 918.72 (481.93 to 1367.03) | 40.73 (20.04 to 61.75) | 888.62 (437.12 to 1347.09) |
| Pakistan | 2519.40 (1620.31 to 3425.22) | 2232.78 (1435.97 to 3035.56) | 3226.98 (2105.53 to 4414.55) | 2269.60 (1480.86 to 3104.84) | 4506.06 (2940.30 to 6128.05) | 2462.21 (1606.65 to 3348.50) | 5748.11 (3747.01 to 7849.72) | 2565.40 (1672.34 to 3503.36) |
| Palau | 0.10  (0.05 to 0.15) | 644.44 (325.51 to 967.64) | 0.11  (0.05 to 0.17) | 570.31 (269.64 to 876.48) | 0.10  (0.05 to 0.15) | 547.80 (267.43 to 836.49) | 87.25 (38.58 to 137.47) | 484.50 (214.26 to 763.42) |
| Palestine | 77.50 (53.53 to 101.74) | 3743.98 (2585.95 to 4915.05) | 94.86 (63.73 to 125.76) | 3109.05 (2088.69 to 4121.78) | 125.55 (85.14 to 167.14) | 3020.76 (2048.57 to 4021.26) | 130.46 (86.55 to 176.14) | 2632.01 (1746.11 to 3553.65) |
| Papua New Guinea | 65.62 (40.64 to 90.36) | 1605.39 (994.27 to 2210.71) | 84.44 (52.87 to 116.83) | 1519.93 (951.73 to 2102.94) | 118.19 (75.27 to 161.28) | 1559.35 (993.09 to 2127.80) | 136.20 (83.63 to 189.29) | 1380.46 (847.58 to 1918.48) |
| Paraguay | 24,94 (10.46 to 39.43) | 616.42 (258.58 to 974.78) | 30.13 (13.24 to 47.91) | 588.23 (258.41 to 935.23) | 29.91 (11.06 to 49.44) | 431.57 (159.58 to 713.36) | 33.90 (13.73 to 52.95) | 555.82 (225.18 to 868.11) |
| Peru | 140.12 (66.07 to 214.44) | 644.81 (304.03 to 986.85) | 146.97 (67.07 to 230.14) | 576.19 (262.92 to 902.21) | 131.22 (51.80 to 212.63) | 453.87 (179.18 to 735.46) | 123.14 (44.41 to 203.67) | 362.22 (130.64 to 599.10) |
| Philippines | 1045.88 (645.75 to 1446.51) | 1652.56 (1020.32 to 2285.57) | 1181.01 (722.59 to 1657.31) | 1479.15 (905.00 to 2075.68) | 1245.64 (746.66 to 1751.03) | 1289.03 (772.67 to 1812.03) | 1068.46 (590.75 to 1569.68) | 952.77 (526.78 to 1399.72) |
| Poland | 300.23 (167.01 to 441.56) | 786.81 (437.68 to 1157.19) | 260.15 (137.40 to 388.24) | 679.37 (358.81 to 1013.88) | 199.67 (97.18 to 307.88) | 520.37 (253.28 to 802.38) | 152.03 (61.30 to 243.54) | 395.56 (159.50 to 633.65) |
| Portugal | 62.16 (25.61 to 98.59) | 613.18 (252.62 to 972.53) | 50.47 (18.29 to 83.74) | 478.67 (173.44 to 794.24) | 45.28 (15.98 to 75.88) | 419.46 (148.03 to 702.94) | 41.38 (14.07 to 68.67) | 388.54 (132.09 to 644.67) |
| Puerto Rico | 9.40  (2.74 to 16.36) | 260.24  (75.74 to 452.80) | 8.31  (2.21 to 14.66) | 214.52  (56.94 to 378.31) | 7.25  (1.87 to 12.85) | 190.77  (49.15 to 338.34) | 6.31  (1.58 to 11.21) | 179.10 (45.00 to 318.25) |
| Qatar | 2.68  (1.08 to 4.32) | 602.46 (242.94 to 971.47) | 3.33  (1.39 to 5.36) | 567.55 (237.48 to 912.65) | 8.05  (2.97 to 13.11) | 464.65 (171.57 to 756.53) | 12.68  (4.67 to 20.92) | 442.52 (162.92 to 730.42) |
| Republic of Korea | 229.54 (78.78 to 377.88) | 517.70 (177.69 to 852.28) | 141.87 (35.50 to 255.43) | 303.25  (75.88 to 545.99) | 77.20 (14.72 to 154.42) | 156.48  (29.83 to 313.00) | 47.53  (6.04 to 105.66) | 89.01  (11.30 to 197.87) |
| Republic of Moldova | 41.96 (22.72 to 61.54) | 943.72 (510.96 to 1384.04) | 63.03 (38.82 to 87.74) | 1503.85 (926.13 to 2093.58) | 48.35 (28.78 to 67.86) | 1248.12 (742.99 to 1751.79) | 34.67 (18.65 to 50.29) | 940.12 (505.59 to 1363.57) |
| Romania | 169.18 (86.61 to 249.06) | 723.10 (370.20 to 1064.51) | 171.17 (91.48 to 254.94) | 763.24 (407.92 to 1136.74) | 111.98 (53.22 to 172.36) | 540.83 (257.04 to 832.44) | 81.42 (34.61 to 129.90) | 423.26 (179.93 to 675.26) |
| Russian Federation | 785.86 (348.38 to 1242.68) | 520.36 (230.68 to 822.84) | 1056.33 (544.24 to 1578.80) | 708.55 (365.06 to 1059.01) | 715.23 (313.93 to 1126.04) | 491.93 (215.92 to 774.48) | 594.41 (225.32 to 969.46) | 405.14 (153.57 to 660.77) |
| Rwanda | 111.30  (65.70 to  158.13) | 1,551.68  (916.02 to  2,204.52) | 145.23  (88.72 to  199.29) | 1,797.41  (1,098.08 to  2,466.50) | 145.95  (84.92 to  203.89) | 1,414.66  (823.10 to  1,976.20) | 128.09  (68.75 to  191.88) | 1,009.52  (541.88 to  1,512.30) |
| Saint Kitts and Navis | 0.18  (0.07 to 288.54) | 442.48 (173.76 to 697.77) | 0.15  (0.05 to 0.26) | 327.04 (108.21 to 555.29) | 0.15  (0.05 to 0.26) | 280.36  (84.43 to 479.05) | 0.15  (0.04 to 0.25) | 243.77 (69.55 to 425.32) |
| Saint Lucia | 0.75  (0.32 to 1.19) | 547.25 (234.40 to 868.24) | 0.70  (0.29 to 1.13) | 448.88 (187.96 to 727.76) | 0.67  (0.27 to 1.10) | 398.79 (160.26 to 651.73) | 0.61  (0.21 to 1.01) | 349.30 (118.45 to 580.67) |
| Saint Vincent and the Grenadines | 0.72  (0.33 to 1.11) | 655.79 (302.91 to 1008.97) | 0.56  (0.24 to 0.89) | 512.15 (215.26 to 810.63) | 0.45  (0.16 to 0.74) | 401.23 (144.62 to 659.45) | 0.40  (0.14 to 0.67) | 357.82 (121.37 to 595.08) |
| Samoa | 1.88  (1.11 to 2.66) | 1148.45 (677.76 to 1625.34) | 1.90  (1.08 to 2.70) | 1094.97 (625.70 to 1561.12) | 1.73  (0.94 to 2.48) | 933.38 (506.94 to 1339.55) | 1.91  (1.06 to 2.79) | 904.95 (502.33 to 1320.13) |
| San Marino | 0.07  (0.02 to 0.13) | 307.48  (84.93 to 543.56) | 0.70  (0.02 to 0.13) | 254.72  (66.60 to 459.61) | 0.07  (0.02 to 0.13) | 217.79  (52.81 to 408.40) | 0.09  (0.02 to 0.16) | 262.77 (69.38 to 476.69) |
| Sao Tome and Principe | 927.90  (377.77 to  1,472.30) | 763.50  (310.84 to  1,211.44) | 1,144.74  (466.47 to  1,809.55) | 799.84  (325.93 to  1,264.36) | 1,239.02  (514.71 to  1,991.79) | 709.56  (294.76 to  1,140.65) | 1,183.76  (420.96 to  1,945.19) | 576.36  (204.96 to  947.09) |
| Saudi Arab Republic | 134.67 (67.55 to 198.58) | 839.35 (420.99 to 1237.61) | 168.39 (83.74 to 252.03) | 805.17 (400.44 to 1205.13) | 199.49 (93.23 to 307.79) | 714.36 (333.87 to 1102.19) | 214.01 (91.17 to 336.98) | 598.93 (255.16 to 943.08) |
| Scotland | 24.13  (8.54 to 40.39) | 473.77 (167.62 to 793.06) | 21.20  (7.07 to 35.92) | 412.02 (137.35 to 698.07) | 19.49  (6.06 to 33.59) | 363.52 (112.96 to 626.48) | 19.09  (6.04 to 32.98) | 345.89 (109.44 to 597.43) |
| Senegal | 60.21  (25.06 to  98.13) | 790.08  (328.86 to  1,287.69) | 79.43  (30.81 to  129.84) | 803.92  (311.80 to  1,314.22) | 91.29  (35.51 to  148.22) | 729.80  (283.88 to  1,184.91) | 100.15  (35.62 to  166.84) | 661.74  (235.34 to  1,102.41) |
| Serbia | 64.25 (31.81 to 96.47) | 683.71 (338.51 to 1026.60) | 76.35 (40.29 to 113.12) | 797.72 (420.97 to 1181.81) | 57.21 (27.46 to 86.54) | 636.09 (305.37 to 962.24) | 49.11 (24.05 to 75.50) | 561.52 (274.95 to 863.17) |
| Seychelles | 0.58  (0.30 to 0.86) | 794.42 (410.97 to 1178.90) | 0.49  (0.24 to 0.76) | 600.95 (289.95 to 926.44) | 0.48  (0.22 to 0.75) | 516.55 (237.52 to 804.82) | 0.41  (0.17 to 0.66) | 402.10 (167.30 to 649.37) |
| Sierra Leone | 42.11  (21.77 to  62.67) | 1,153.05  (596.03 to  1,716.10) | 60.76  (33.26 to  88.76) | 1,386.55  (758.86 to  2,025.46) | 87.02  (49.57 to  126.01) | 1,367.00  (778.68 to  1,979.44) | 96.56  (51.89 to  143.22) | 1,165.56  (626.30 to  1,728.68) |
| Singapore | 9.40  (2.10 to 16.89) | 308.60  (68.91 to 554.16) | 9.16  (1.51 to 17.38) | 227.35  (37.46 to 431.30) | 6.65  (0.70 to 14.17) | 130.92  (13.72 to 278.99) | 4.29  (0.26 to 10.49) | 75.64  (4.63 to 185.17) |
| Slovakia | 34.49 (18.12 to 51.54) | 652.85 (343.05 to 975.69) | 34.29 (18.06 to 51.20) | 636.25 (335.14 to 950.10) | 24.84 (11.38 to 38.91) | 459.27 (210.45 to 719.39) | 19.71  (7.56 to 31.65) | 362.42 (139.07 to 582.04) |
| Slovenia | 9.88  (4.42 to 15.37) | 501.10 (224.38 to 779.85) | 9.45  (4.24 to 14.80) | 474.33 (212.64 to 743.00) | 7.06  (2.57 to 11.66) | 346.00 (126.12 to 571.47) | 6.56  (2.33 to 11.00) | 316.45 (112.22 to 530.48) |
| Solomon Islands | 6.55  (4.24 to 8.91) | 1923.86 (1245.57 to 2617.85) | 8.04  (5.14 to 11.00) | 1789.46 (1144.30 to 2450.38) | 10.86  (7.10 to 14.61) | 1957.82 (1280.09 to 2634.42) | 11.63  (7.47 to 15.90) | 1773.21 (1139.87 to 2425.57) |
| Somalia | 213.15  (136.80 to  287.27) | 2,982.21  (1,913.90 to  4,019.11) | 375.58  (245.89 to  507.61) | 3,595.28  (2,353.83 to  4,859.13) | 546.69  (362.83 to  741.45) | 3,709.39  (2,461.84 to  5,030.80) | 746.53  (487.12 to  1,005.94) | 3,669.70  (2,394.54 to  4,944.88) |
| South Africa | 151.72  (53.27 to  253.60) | 411.95  (144.65 to  688.59) | 190.18  (70.33 to  315.08) | 418.21  (154.65 to  692.85) | 180.70  (61.80 to  306.24) | 356.22  (121.82 to  603.69) | 180.73  (57.06 to  309.21) | 325.12  (102.65 to  556.26) |
| South Sudan | 18.85  (4.37 to  35.27) | 321.67  (74.53 to  601.99) | 25.68  (6.10 to 47.91) | 354.67  (84.22 to  661.77) | 30.18  (7.02 to 55.19) | 322.74  (75.06 to  590.17) | 26.63  (4.63 to 52.04) | 286.91  (49.91 to  560.57) |
| Spain | 641.15 (390.89 to 894.90) | 1653.25 (1007.94 to 2307.56) | 574.54 (339.33 to 805.29) | 1407.84 (831.49 to 1973.25) | 557.72 (315.49 to 802.37) | 1187.25 (671.60 to 1708.05) | 523.73 (292.63 to 757.54) | 1138.02 (635.86 to 1646.07) |
| Sri Lanka | 282.11 (174.04 to 392.89) | 1638.14 (1010.57 to 2281.36) | 234.67 (143.61 to 331.75) | 1249.27 (764.53 to 1766.10) | 190.97 (103.17 to 277.67) | 931.50 (503.21 to 1354.36) | 138.07 (65.34 to 211.01) | 631.77 (299.00 to 965.55) |
| Suriname | 1.80  (0.68 to 2.93) | 466.16 (175.65 to 756.77) | 2.09  (0.76 to 3.38) | 465.94 (169.65 to 751.59) | 1.99  (0.68 to 3.37) | 369.46 (126.38 to 626.16) | 1.87  (0.62 to 3.18) | 324.79 (108.38 to 551.64) |
| Sweden | 46.18 (19.99 to 72.89) | 537.65 (232.72 to 848.68) | 47.44 (20.64 to 75.05) | 532.74 (231.75 to 842.85) | 43.90 (17.68 to 71.98) | 465.76 (187.58 to 763.62) | 52.05 (22.07 to 83.60) | 509.17 (215.90 to 817.76) |
| Switzerland | 21.66  (6.24 to 38.01) | 315.45  (90.92 to 553.57) | 22.12  (6.51 to 39.32) | 303.04  (89.16 to 538.74) | 21.75  (5.84 to 38.89) | 273.28  (73.40 to 488.59) | 22.39  (5.87 to 40.29) | 255.20 (66.85 to 459.11) |
| Taiwan (Province of China) | 132.55 (73.75 to 193.61) | 649.79 (361.53 to 949.10) | 97.46 (48.00 to 149.03) | 437.48 (215.48 to 668.98) | 73.70 (32.79 to 116.49) | 317.89 (141.45 to 502.44) | 62.31 (24.46 to 100.60) | 263.80 (103.57 to 425.91) |
| Tajikistan | 67.12 (39.31 to 96.12) | 1248.63 (731.27 to 1788.12) | 141.06 (90.04 to 192.31) | 2218.89 (1416.33 to 3025.06) | 146.19 (92.37 to 201.06) | 1882.44 (1189.51 to 2589.10) | 146.36 (89.20 to 203.70) | 1541.87 (939.72 to 2145.90) |
| Thailand | 1164.08  (761.87 to 1574.34) | 2046.70  (1339.53 to 2768.03) | 914.39  (564.73 to 1262.14) | 1458.23  (900.61 to 2012.82) | 722.02  (413.87 to 1038.41) | 1,066.88  (611.55 to 1534.40) | 547.04 (290.73 to 827.30) | 780.24  (414.66 to 1179.97) |
| Timor-Leste | 17.61  (11.28 to 24.48) | 2,249.53  (1440.37 to 3126.65) | 16.77  (10.40 to 23.40) | 1,843.94  (1,143.47 to 2,572.73) | 12.86  (7.32 to 18.52) | 1,162.78  (662.28 to 1,674.90) | 15.13  (8.38 to 21.79) | 1,133.58  (627.48 to 1,632.48) |
| Togo | 40.91  (20.44 to  61.68) | 1,116.83  (557.99 to  1,683.78) | 56.18  (29.85 to  84.00) | 1,152.32  (612.21 to  1,723.08) | 78.40  (41.93 to  117.26) | 1,227.92  (656.73 to  1,836.60) | 82.07  (41.22 to  125.33) | 1,036.01  (520.29 to  1,582.08) |
| Tokelau | 0.02  (0.01 to 0.03) | 1207.19 (736.33 to 1701.87) | 0.02  (0.01 to 0.03) | 1131.92 (685.56 to 1600.45) | 0.01  (0.01 to 0.02) | 914.35 (508.02 to 1312.69) | 0.01  (0.01 to 0.02) | 766.88 (394.47 to 1149.66) |
| Tonga | 1.09  (0.63 to 1.57) | 1131.15 (655.78 to 1617.43) | 1.03  (0.57 to 1.51) | 1020.83 (563.13 to 1493.14) | 1.04  (0.57 to 1.51) | 980.53 (534.49 to 1425.72) | 0.92  (0.50 to 1.35) | 900.07 (487.90 to 1317.80) |
| Trinidad and Tobago | 4.85  (1.75 to 8.01) | 403.37 (145.71 to 665.95) | 4.72  (1.56 to 7.93) | 365.14 (120.85 to 613.19) | 3.21  (0.95 to 5.63) | 238.58  (70.59 to 418.12) | 3.03  (0.83 to 5.32) | 218.33 (59.76 to 383.50) |
| Tunisia | 220.19 (145.00 to 292.59) | 2609.13 (1718.17 to 3466.99) | 217.50 (141.41 to 291.78) | 2196.99 (1428.37 to 2947.27) | 196.62 (126.26 to 267.55) | 1816.66 (1166.64 to 2472.05) | 186.10 (116.27 to 259.69) | 1608.21 (1004.75 to 2244.17) |
| Turkey | 1141.43 (730.88 to 1574.59) | 1909.61 (1222.76 to 2634.30) | 1107.27 (676.87 to 1572.63) | 1598.50 (977.17 to 2270.33) | 996.41 (572.12 to 1414.01) | 1332.71 (765.21 to 1891.26) | 825.13 (419.10 to 1237.20) | 1014.18 (515.12 to 1520.65) |
| Turkmenistan | 38.62 (22.89 to 54.41) | 1042.27 (617.67 to 1468.59) | 58.69 (37.11 to 81.54) | 1390.74 (879.48 to 1932.18) | 41.40 (23.34 to 59.80) | 903.31 (509.29 to 1304.63) | 28.98 (13.53 to 44.77) | 570.11 (266.25 to 880.75) |
| Tuvalu | 0.16  (0.10 to 0.21) | 1682.01 (1098.89 to 2270.22) | 0.13  (0.08 to 0.18) | 1364.18 (872.33 to 1875.37) | 0.14  (0.09 to 0.19) | 1296.85 (810.12 to 1806.36) | 0.14  (0.09 to 0.20) | 1198.96 (725.40 to 1676.94) |
| Uganda | 293.91  (173.89 to  419.03) | 1,697.38  (1,004.24 to  2,419.97) | 368.00  (205.81 to  529.73) | 1,515.88  (847.78 to 2,182.11) | 397.99  (216.84 to 598.46) | 1,221.95  (665.76 to  1,837.47) | 412.16  (203.85 to  619.44) | 1,002.38  (495.77 to  1,506.50) |
| Ukraine | 334.32 (155.77 to 511.72) | 634.82 (295.78 to 971.69) | 474.28 (267.41 to 686.43) | 945.84 (538.36 to 1381.94) | 332.80 (166.78 to 503.74) | 719.26 (360.45 to 1088.71) | 299.41 (150.89 to 455.80) | 679.83 (342.60 to 1034.92) |
| United Arab Emirates | 10.19  (4.45 to 16.23) | 544.11 (237.79 to 867.11) | 17.34  (7.24 to 27.62) | 537.28 (224.33 to 855.63) | 51.60 (21.61 to 82.23) | 611.65 (256.12 to 974.65) | 57.09 (24.96 to 89.67) | 617.77 (270.05 to 970.32) |
| United Republic of Tanzania | 330.93  (181.03 to  482.79) | 1,277.67  (698.94 to  1,863.96) | 451.87  (257.21 to  660.63) | 1,315.71  (748.93 to  1,923.57) | 478.23  (260.82 to  709.59) | 1,069.74  (583.41 to  1,587.27) | 465.04  (218.73 to  716.10) | 819.66  (385.52 to  1,262.16) |
| United States of America | 1322.16 (503.59 to 2162.56) | 521.34 (198.57 to 852.73) | 1389.85 (524.36 to 2280.83) | 495.17 (186.82 to 812.60) | 1345.43 (459.64 to 2268.84) | 435.46 (148.77 to 734.34) | 1316.65 (424.28 to 2243.98) | 401.44 (129.36 to 684.18) |
| United States Virgin Islands | 0.30  (0.09 to 0.51) | 282.67  (85.40 to 385.22) | 0.22  (0.06 to 0.39) | 195.99  (50.24 to 347.27) | 0.15  (0.03 to 0.28) | 140.31  (30.31 to 259.36) | 0.16  (0.04 to 0.29) | 154.71 (35.05 to 280.17) |
| Uruguay | 22.60 (10.41 to 35.05) | 720.01 (331.53 to 1116.46) | 19.13  (8.12 to 30.82) | 580.13 (246.07 to 934.38) | 19.10  (7.58 to 31.02) | 567.71 (225.30 to 922.21) | 15.65  (5.77 to 26.19) | 455.52 (168.01 to 762.14) |
| Uzbekistan | 305.20 (187.05 to 425.26) | 1456.95 (892.93 to 2030.10) | 420.05 (270.69 to 575.22) | 1666.41 (1073.87 to 2282.01) | 393.90 (238.21 to 549.31) | 1350.97 (816.98 to 1883.97) | 334.42 (188.58 to 481.67) | 990.04 (559.96 to 1430.25) |
| Vanuatu | 2.33  (1.44 to 3.23) | 1539.66 (949.90 to 2135.54) | 2.88  (1.81 to 3.98) | 1497.28 (939.33 to 2067.45) | 3.60  (2.22 to 4.97) | 1463.06 (902.72 to 2018.85) | 4.21  (2.65 to 5.88) | 1429.79 (898.27 to 1994.92) |
| Viet Nam | 1455.54  (932.82 to 1,963.47) | 2,142.32  (1,372.96 to 2,889.92) | 1,291.05  (819.28 to 1,778.63) | 1,607.74  (1,020.25 to 2,214.91) | 1,023.32  (626.36 to 1,436.55) | 1,143.75  (700.07 to 1,605.62) | 763.59  (429.77 to 1,105.42) | 792.33  (445.95 to 1,147.03) |
| Wales | 17.10  (6.92 to 27.25) | 591.02 (238.96 to 941.64) | 14.61  (5.61 to 24.08) | 495.03 (190.20 to 815.77) | 12.18  (3.92 to 20.88) | 395.02 (127.04 to 677.01) | 11.76  (3.56 to 20.43) | 369.00 (111.59 to 641.03) |
| Yemen | 524.18 (349.44 to 704.19) | 3818.01 (2545.29 to 5129.20) | 662.78 (432.68 to 894.87) | 3545.84 (2314.78 to 4787.50) | 760.22 (484.58 to 1042.14) | 3017.16 (1923.21 to 4136.05) | 1188.92 (786.61 to 1599.81) | 3774.01 (2496.95 to 5078.30) |
| Zambia | 74.21  (36.07 to  113.55) | 934.22  (454.15 to  1,429.56) | 107.15  (52.34 to  162.26) | 1,082.09  (528.56 to  1,638.61) | 117.03  (56.00 to  182.83) | 859.25  (411.18 to  1,342.37) | 118.79  (46.29 to  194.49) | 651.34  (253.82 to  1,066.42) |
| Zimbabwe | 83.81  (36.78 to 128.09) | 810.73  (355.74 to  1,239.01) | 89.76  (40.98 to  142.57) | 750.87  (342.77 to  1,192.57) | 148.26  (76.03 to  221.63) | 1,138.01  (583.57 to  1,701.16) | 148.72  (77.18 to  224.79) | 990.76  (514.16 to  1,497.48) |

**Table 2: APC values (with lower and upper 95 CI) based on 30-year data for each country/territory**

| **Countries** | **APC** | **Lower** | **Upper** |
| --- | --- | --- | --- |
| Afghanistan | -0.22 | -0.74 | 0.29 |
| Albania | -2.51 | -2.76 | -2.26 |
| Algeria | -1.20 | -1.34 | -1.06 |
| American Samoa | 0.34 | 0.28 | 0.40 |
| Andorra | -0.71 | -0.85 | -0.58 |
| Angola | -2.46 | -2.92 | -2.00 |
| Antigua and Barbuda | -1.13 | -1.21 | -1.06 |
| Argentina | -1.38 | -1.49 | -1.26 |
| Armenia | -2.43 | -2.93 | -1.92 |
| Australia | -0.11 | -0.33 | 0.11 |
| Austria | -1.35 | -1.40 | -1.30 |
| Azerbaijan | -2.41 | -3.31 | -1.51 |
| Bahamas | -0.34 | -0.45 | -0.23 |
| Baharain | -1.39 | -1.42 | -1.35 |
| Bangladesh | -5.59 | -5.96 | -5.21 |
| Barbados | -0.56 | -0.66 | -0.46 |
| Belarus | -2.30 | -2.76 | -1.83 |
| Belgium | -1.20 | -1.27 | -1.14 |
| Belize | -1.38 | -1.51 | -1.24 |
| Benin | -0.82 | -0.96 | -0.68 |
| Bermuda | -1.47 | -1.58 | -1.36 |
| Bhutan | -2.41 | -2.43 | -2.39 |
| Bolivia (Plurinational State of) | -1.75 | -1.88 | -1.63 |
| Bosnia and Herzegovina | -3.79 | -4.10 | -3.47 |
| Botswana | -1.68 | -1.73 | -1.63 |
| Brazil | -1.63 | -1.73 | -1.53 |
| Brunei Darussalam | -2.11 | -2.48 | -1.74 |
| Bulgaria | -1.93 | -2.27 | -1.59 |
| Burkina Faso | -0.83 | -0.92 | -0.73 |
| Burundi | 0.47 | 0.25 | 0.69 |
| Cabo Verde | -2.66 | -2.74 | -2.58 |
| Cambodia | -2.80 | -3.06 | -2.54 |
| Cameroon | -0.34 | -0.60 | -0.08 |
| Canada | 2.05 | 1.48 | 2.62 |
| Caribbean | -0.36 | -0.46 | -0.25 |
| Central African Republic | 0.63 | 0.57 | 0.68 |
| Chad | -1.31 | -1.52 | -1.11 |
| Chile | -2.42 | -2.61 | -2.23 |
| China | -2.75 | -2.86 | -2.65 |
| Colombia | -2.08 | -2.23 | -1.92 |
| Comoros | -0.34 | -0.48 | -0.21 |
| Congo | -0.52 | -0.69 | -0.36 |
| Cook Islands | -1.74 | -1.81 | -1.66 |
| Costa Rica | -1.50 | -1.57 | -1.42 |
| Cote DIvoire | -0.13 | -0.38 | 0.13 |
| Croatia | -1.51 | -1.65 | -1.38 |
| Cuba | -0.88 | -1.31 | -0.44 |
| Cyprus | -1.34 | -1.50 | -1.18 |
| Czechia | -1.90 | -2.00 | -1.81 |
| Democratic People’s Republic of Korea | 0.92 | 0.76 | 1.09 |
| Democratic Republic of Congo | 0.94 | 0.33 | 1.56 |
| Denmark | -1.62 | -1.67 | -1.58 |
| Djibouti | -0.62 | -0.95 | -0.29 |
| Dominica | -1.42 | -1.50 | -1.35 |
| Dominican Republic | -2.26 | -2.31 | -2.20 |
| Ecuador | -1.38 | -1.47 | -1.29 |
| Egypt | -1.66 | -1.69 | -1.63 |
| EI Salvado | -1.96 | -2.03 | -1.89 |
| England | -1.23 | -1.33 | -1.14 |
| Equatorial Guinea | -7.92 | -8.92 | -6.92 |
| Eritrea | -1.18 | -1.52 | -0.84 |
| Estonia | -2.52 | -2.75 | -2.29 |
| Eswatini | -1.07 | -1.15 | -1.00 |
| Ethiopia | -1.83 | -2.28 | -1.37 |
| Fiji | -0.88 | -0.92 | -0.83 |
| Finland | -1.30 | -1.43 | -1.18 |
| France | -1.17 | -1.25 | -1.09 |
| Gabon | 0.16 | 0.09 | 0.23 |
| Gambia | -0.07 | -0.13 | -0.01 |
| Georgia | -1.26 | -2.04 | -0.48 |
| Germany | -1.43 | -1.50 | -1.36 |
| Ghana | -1.50 | -1.69 | -1.31 |
| Greece | -1.55 | -1.78 | -1.31 |
| Greenland | 0.60 | 0.32 | 0.87 |
| Grenada | -2.17 | -2.28 | -2.05 |
| Guam | -0.48 | -0.54 | -0.41 |
| Guatemala | -1.44 | -1.52 | -1.35 |
| Guinea | -0.10 | -0.27 | 0.08 |
| Guinea-Bissau | -0.15 | -0.32 | 0.02 |
| Guyana | -1.96 | -2.04 | -1.88 |
| Haiti | 0.05 | -0.05 | 0.15 |
| Honduras | -1.01 | -1.14 | -0.88 |
| Hungary | -1.79 | -1.93 | -1.66 |
| Iceland | -1.33 | -1.41 | -1.24 |
| India | -0.65 | -0.69 | -0.60 |
| Indonesia | -2.22 | -2.41 | -2.03 |
| Iran | -2.10 | -2.19 | -2.01 |
| Iraq | -1.20 | -1.59 | -0.81 |
| Ireland | -2.87 | -3.11 | -2.62 |
| Israel | -1.17 | -1.22 | -1.11 |
| Italy | -0.73 | -0.83 | -0.63 |
| Jamaica | -0.70 | -0.77 | -0.62 |
| Japan | -1.87 | -1.99 | -1.76 |
| Jordan | -1.35 | -1.52 | -1.18 |
| Kazakhstan | -2.08 | -2.58 | -1.58 |
| Kenya | -0.60 | -0.81 | -0.38 |
| Kiribati | 0.15 | 0.09 | 0.21 |
| Kuwait | -1.37 | -1.54 | -1.20 |
| Kyrgyzstan | 0.02 | -0.42 | 0.47 |
| Lao People’s Democratic Republic | -3.02 | -3.27 | -2.77 |
| Latvia | -2.32 | -2.75 | -1.88 |
| Lebanon | -1.56 | -1.65 | -1.46 |
| Lesotho | -1.96 | -2.05 | -1.87 |
| Liberia | -0.52 | -1.10 | 0.06 |
| Libya | 0.26 | -0.06 | 0.58 |
| Lithuania | -2.72 | -3.14 | -2.30 |
| Luxembourg | -1.59 | -1.68 | -1.49 |
| Madagascar | -0.15 | -0.29 | -0.01 |
| Malawi | -0.65 | -0.77 | -0.52 |
| Malaysia | -2.74 | -2.84 | -2.64 |
| Maldives | -3.53 | -3.62 | -3.43 |
| Mali | -0.94 | -1.00 | -0.87 |
| Malta | -2.06 | -2.14 | -1.97 |
| Marshall Islands | -0.58 | -0.65 | -0.51 |
| Mauritania | -0.99 | -1.09 | -0.90 |
| Mauritius | -2.93 | -3.05 | -2.82 |
| Mexico | -1.04 | -1.08 | -1.01 |
| Micronesia (Federated States of) | -0.46 | -0.48 | -0.43 |
| Monaco | -1.21 | -1.29 | -1.13 |
| Mongolia | -2.35 | -2.74 | -1.96 |
| Montenegro | -1.18 | -1.62 | -0.74 |
| Morocco | -1.49 | -1.59 | -1.40 |
| Mozambique | -2.10 | -2.22 | -1.98 |
| Myanmar | -3.96 | -4.29 | -3.63 |
| Namibia | -1.15 | -1.28 | -1.01 |
| Nauru | 1.31 | 0.45 | 2.19 |
| Nepal | -1.34 | -1.42 | -1.27 |
| Netherlands | -1.43 | -1.52 | -1.33 |
| New Zealand | 0.00 | -0.19 | 0.20 |
| Niger | 0.48 | 0.32 | 0.64 |
| Nigeria | -1.75 | -2.12 | -1.38 |
| Niue | -1.25 | -1.34 | -1.17 |
| North Macedonia | -1.94 | -2.23 | -1.64 |
| Northern Ireland | -1.38 | -1.48 | -1.28 |
| Northern Mariana Islands | 0.73 | 0.58 | 0.89 |
| Norway | -1.16 | -1.22 | -1.10 |
| Oman | -1.55 | -1.66 | -1.45 |
| Pakistan | 0.60 | 0.56 | 0.65 |
| Palau | -0.82 | -0.90 | -0.75 |
| Palestine | -1.09 | -1.19 | -0.99 |
| Papua New Guinea | -0.29 | -0.42 | -0.17 |
| Paraguay | -1.01 | -1.23 | -0.80 |
| Peru | -2.21 | -2.35 | -2.07 |
| Phillippines | -1.77 | -1.96 | -1.57 |
| Poland | -2.57 | -2.69 | -2.44 |
| Portugal | -1.45 | -1.57 | -1.32 |
| Puerto Rico | -1.27 | -1.41 | -1.12 |
| Qatar | -1.60 | -1.79 | -1.41 |
| Republic of Korea | -5.48 | -5.67 | -5.28 |
| Republic of Moldova | -0.35 | -1.01 | 0.32 |
| Romania | -2.25 | -2.55 | -1.94 |
| Russian Federation | -1.48 | -2.02 | -0.92 |
| Rwanda | -1.77 | -2.18 | -1.36 |
| Saint Kitts and Navis | -1.85 | -1.99 | -1.71 |
| Saint Lucia | -1.35 | -1.41 | -1.29 |
| Saint Vincent and the Grenadines | -2.08 | -2.18 | -1.97 |
| Samoa | -1.04 | -1.14 | -0.93 |
| San Marino | -0.83 | -1.17 | -0.49 |
| Sao Tome and Principe | -1.06 | -1.28 | -0.84 |
| Saudi Arab Republic | -1.17 | -1.34 | -0.99 |
| Scotland | -1.15 | -1.24 | -1.06 |
| Senegal | -0.70 | -0.78 | -0.61 |
| Serbia | -1.10 | -1.36 | -0.83 |
| Seychelles | -2.11 | -2.21 | -2.00 |
| Sierra Leone | 0.11 | -0.27 | 0.49 |
| Singapore | -4.51 | -4.78 | -4.23 |
| Slovakia | -2.39 | -2.62 | -2.16 |
| Slovenia | -2.07 | -2.26 | -1.88 |
| Solomon Islands | -0.01 | -0.16 | 0.14 |
| Somalia | 0.66 | 0.48 | 0.84 |
| South Africa | -1.07 | -1.20 | -0.93 |
| South Sudan | -0.65 | -0.85 | -0.45 |
| Spain | -1.33 | -1.46 | -1.20 |
| Sri Lanka | -3.24 | -3.41 | -3.07 |
| Suriname | -1.58 | -1.76 | -1.40 |
| Sweden | -0.83 | -0.97 | -0.68 |
| Switzerland | -0.82 | -0.90 | -0.75 |
| Taiwan (Province of China) | -3.06 | -3.19 | -2.92 |
| Tajikistan | 0.31 | -0.38 | 1.01 |
| Thailand | -3.17 | -3.26 | -3.08 |
| Timor-Leste | -2.92 | -3.23 | -2.61 |
| Togo | -0.08 | -0.26 | 0.11 |
| Tokelau | -1.71 | -1.82 | -1.60 |
| Tonga | -0.63 | -0.68 | -0.58 |
| Trinidad and Tobago | -2.82 | -3.07 | -2.57 |
| Tunisia | -1.77 | -1.81 | -1.73 |
| Turkey | -2.09 | -2.20 | -1.99 |
| Turkmenistan | -2.58 | -3.26 | -1.89 |
| Tuvalu | -0.90 | -1.04 | -0.76 |
| Uganda | -1.99 | -2.10 | -1.88 |
| Ukraine | -0.34 | -0.87 | 0.19 |
| United Arab Emirates | 0.74 | 0.55 | 0.94 |
| United Republic of Tanzania | -1.75 | -1.98 | -1.51 |
| United States of America | -0.93 | -1.00 | -0.85 |
| United States Virgin Islands | -2.06 | -2.42 | -1.70 |
| Uruguay | -1.36 | -1.53 | -1.20 |
| Uzbekistan | -1.55 | -1.94 | -1.15 |
| Vanuatu | -0.27 | -0.30 | -0.24 |
| Viet Nam | -3.43 | -3.53 | -3.34 |
| Wales | -1.79 | -1.90 | -1.68 |
| Yemen | -0.63 | -0.90 | -0.37 |
| Zambia | -1.58 | -2.01 | -1.15 |
| Zimbabwe | 1.44 | 1.08 | 1.81 |
