# Supplementary material for: Significant regional inequalities in the prevalence of intellectual disability and trends from 1990 to 2019: a systematic analysis of GBD 2019
Source: Epidemiol Psychiatr Sci. 2022 Dec 21;31:e91. doi: 10.1017/S2045796022000701 (PMC9805697; doi:10.1017/S2045796022000701)
Supplement: Supplementary file 1 [file epssup.zip › S2045796022000701sup002.docx]

**Figure 2: Annual Percentage Change (APC) by country & territory across 30 years**


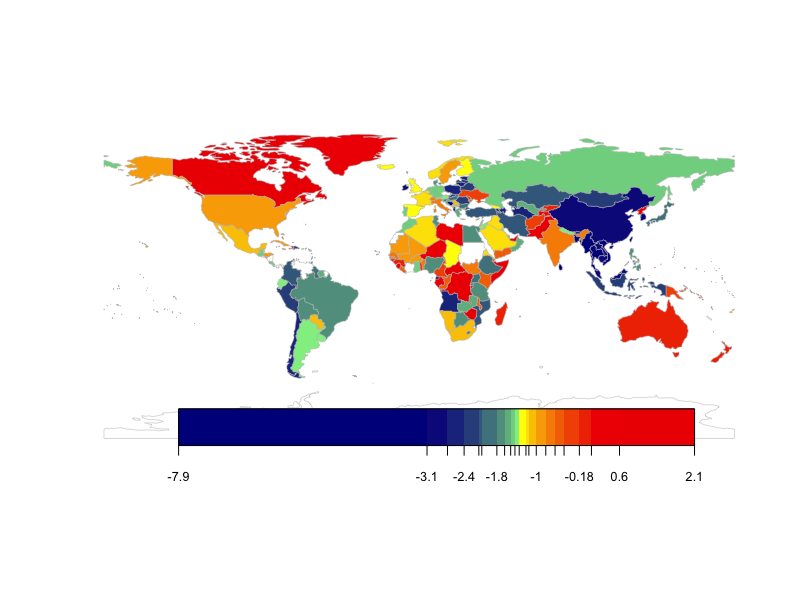


**Table 2: APC values (with lower and upper 95 CI) based on 30-year data for each country/territory**

| **Countries** | **APC** | **Lower** | **Upper** |
| --- | --- | --- | --- |
| Afghanistan | -0.22 | -0.74 | 0.29 |
| Albania | -2.51 | -2.76 | -2.26 |
| Algeria | -1.20 | -1.34 | -1.06 |
| American Samoa | 0.34 | 0.28 | 0.40 |
| Andorra | -0.71 | -0.85 | -0.58 |
| Angola | -2.46 | -2.92 | -2.00 |
| Antigua and Barbuda | -1.13 | -1.21 | -1.06 |
| Argentina | -1.38 | -1.49 | -1.26 |
| Armenia | -2.43 | -2.93 | -1.92 |
| Australia | -0.11 | -0.33 | 0.11 |
| Austria | -1.35 | -1.40 | -1.30 |
| Azerbaijan | -2.41 | -3.31 | -1.51 |
| Bahamas | -0.34 | -0.45 | -0.23 |
| Baharain | -1.39 | -1.42 | -1.35 |
| Bangladesh | -5.59 | -5.96 | -5.21 |
| Barbados | -0.56 | -0.66 | -0.46 |
| Belarus | -2.30 | -2.76 | -1.83 |
| Belgium | -1.20 | -1.27 | -1.14 |
| Belize | -1.38 | -1.51 | -1.24 |
| Benin | -0.82 | -0.96 | -0.68 |
| Bermuda | -1.47 | -1.58 | -1.36 |
| Bhutan | -2.41 | -2.43 | -2.39 |
| Bolivia (Plurinational State of) | -1.75 | -1.88 | -1.63 |
| Bosnia and Herzegovina | -3.79 | -4.10 | -3.47 |
| Botswana | -1.68 | -1.73 | -1.63 |
| Brazil | -1.63 | -1.73 | -1.53 |
| Brunei Darussalam | -2.11 | -2.48 | -1.74 |
| Bulgaria | -1.93 | -2.27 | -1.59 |
| Burkina Faso | -0.83 | -0.92 | -0.73 |
| Burundi | 0.47 | 0.25 | 0.69 |
| Cabo Verde | -2.66 | -2.74 | -2.58 |
| Cambodia | -2.80 | -3.06 | -2.54 |
| Cameroon | -0.34 | -0.60 | -0.08 |
| Canada | 2.05 | 1.48 | 2.62 |
| Caribbean | -0.36 | -0.46 | -0.25 |
| Central African Republic | 0.63 | 0.57 | 0.68 |
| Chad | -1.31 | -1.52 | -1.11 |
| Chile | -2.42 | -2.61 | -2.23 |
| China | -2.75 | -2.86 | -2.65 |
| Colombia | -2.08 | -2.23 | -1.92 |
| Comoros | -0.34 | -0.48 | -0.21 |
| Congo | -0.52 | -0.69 | -0.36 |
| Cook Islands | -1.74 | -1.81 | -1.66 |
| Costa Rica | -1.50 | -1.57 | -1.42 |
| Cote DIvoire | -0.13 | -0.38 | 0.13 |
| Croatia | -1.51 | -1.65 | -1.38 |
| Cuba | -0.88 | -1.31 | -0.44 |
| Cyprus | -1.34 | -1.50 | -1.18 |
| Czechia | -1.90 | -2.00 | -1.81 |
| Democratic People’s Republic of Korea | 0.92 | 0.76 | 1.09 |
| Democratic Republic of Congo | 0.94 | 0.33 | 1.56 |
| Denmark | -1.62 | -1.67 | -1.58 |
| Djibouti | -0.62 | -0.95 | -0.29 |
| Dominica | -1.42 | -1.50 | -1.35 |
| Dominican Republic | -2.26 | -2.31 | -2.20 |
| Ecuador | -1.38 | -1.47 | -1.29 |
| Egypt | -1.66 | -1.69 | -1.63 |
| EI Salvado | -1.96 | -2.03 | -1.89 |
| England | -1.23 | -1.33 | -1.14 |
| Equatorial Guinea | -7.92 | -8.92 | -6.92 |
| Eritrea | -1.18 | -1.52 | -0.84 |
| Estonia | -2.52 | -2.75 | -2.29 |
| Eswatini | -1.07 | -1.15 | -1.00 |
| Ethiopia | -1.83 | -2.28 | -1.37 |
| Fiji | -0.88 | -0.92 | -0.83 |
| Finland | -1.30 | -1.43 | -1.18 |
| France | -1.17 | -1.25 | -1.09 |
| Gabon | 0.16 | 0.09 | 0.23 |
| Gambia | -0.07 | -0.13 | -0.01 |
| Georgia | -1.26 | -2.04 | -0.48 |
| Germany | -1.43 | -1.50 | -1.36 |
| Ghana | -1.50 | -1.69 | -1.31 |
| Greece | -1.55 | -1.78 | -1.31 |
| Greenland | 0.60 | 0.32 | 0.87 |
| Grenada | -2.17 | -2.28 | -2.05 |
| Guam | -0.48 | -0.54 | -0.41 |
| Guatemala | -1.44 | -1.52 | -1.35 |
| Guinea | -0.10 | -0.27 | 0.08 |
| Guinea-Bissau | -0.15 | -0.32 | 0.02 |
| Guyana | -1.96 | -2.04 | -1.88 |
| Haiti | 0.05 | -0.05 | 0.15 |
| Honduras | -1.01 | -1.14 | -0.88 |
| Hungary | -1.79 | -1.93 | -1.66 |
| Iceland | -1.33 | -1.41 | -1.24 |
| India | -0.65 | -0.69 | -0.60 |
| Indonesia | -2.22 | -2.41 | -2.03 |
| Iran | -2.10 | -2.19 | -2.01 |
| Iraq | -1.20 | -1.59 | -0.81 |
| Ireland | -2.87 | -3.11 | -2.62 |
| Israel | -1.17 | -1.22 | -1.11 |
| Italy | -0.73 | -0.83 | -0.63 |
| Jamaica | -0.70 | -0.77 | -0.62 |
| Japan | -1.87 | -1.99 | -1.76 |
| Jordan | -1.35 | -1.52 | -1.18 |
| Kazakhstan | -2.08 | -2.58 | -1.58 |
| Kenya | -0.60 | -0.81 | -0.38 |
| Kiribati | 0.15 | 0.09 | 0.21 |
| Kuwait | -1.37 | -1.54 | -1.20 |
| Kyrgyzstan | 0.02 | -0.42 | 0.47 |
| Lao People’s Democratic Republic | -3.02 | -3.27 | -2.77 |
| Latvia | -2.32 | -2.75 | -1.88 |
| Lebanon | -1.56 | -1.65 | -1.46 |
| Lesotho | -1.96 | -2.05 | -1.87 |
| Liberia | -0.52 | -1.10 | 0.06 |
| Libya | 0.26 | -0.06 | 0.58 |
| Lithuania | -2.72 | -3.14 | -2.30 |
| Luxembourg | -1.59 | -1.68 | -1.49 |
| Madagascar | -0.15 | -0.29 | -0.01 |
| Malawi | -0.65 | -0.77 | -0.52 |
| Malaysia | -2.74 | -2.84 | -2.64 |
| Maldives | -3.53 | -3.62 | -3.43 |
| Mali | -0.94 | -1.00 | -0.87 |
| Malta | -2.06 | -2.14 | -1.97 |
| Marshall Islands | -0.58 | -0.65 | -0.51 |
| Mauritania | -0.99 | -1.09 | -0.90 |
| Mauritius | -2.93 | -3.05 | -2.82 |
| Mexico | -1.04 | -1.08 | -1.01 |
| Micronesia (Federated States of) | -0.46 | -0.48 | -0.43 |
| Monaco | -1.21 | -1.29 | -1.13 |
| Mongolia | -2.35 | -2.74 | -1.96 |
| Montenegro | -1.18 | -1.62 | -0.74 |
| Morocco | -1.49 | -1.59 | -1.40 |
| Mozambique | -2.10 | -2.22 | -1.98 |
| Myanmar | -3.96 | -4.29 | -3.63 |
| Namibia | -1.15 | -1.28 | -1.01 |
| Nauru | 1.31 | 0.45 | 2.19 |
| Nepal | -1.34 | -1.42 | -1.27 |
| Netherlands | -1.43 | -1.52 | -1.33 |
| New Zealand | 0.00 | -0.19 | 0.20 |
| Niger | 0.48 | 0.32 | 0.64 |
| Nigeria | -1.75 | -2.12 | -1.38 |
| Niue | -1.25 | -1.34 | -1.17 |
| North Macedonia | -1.94 | -2.23 | -1.64 |
| Northern Ireland | -1.38 | -1.48 | -1.28 |
| Northern Mariana Islands | 0.73 | 0.58 | 0.89 |
| Norway | -1.16 | -1.22 | -1.10 |
| Oman | -1.55 | -1.66 | -1.45 |
| Pakistan | 0.60 | 0.56 | 0.65 |
| Palau | -0.82 | -0.90 | -0.75 |
| Palestine | -1.09 | -1.19 | -0.99 |
| Papua New Guinea | -0.29 | -0.42 | -0.17 |
| Paraguay | -1.01 | -1.23 | -0.80 |
| Peru | -2.21 | -2.35 | -2.07 |
| Phillippines | -1.77 | -1.96 | -1.57 |
| Poland | -2.57 | -2.69 | -2.44 |
| Portugal | -1.45 | -1.57 | -1.32 |
| Puerto Rico | -1.27 | -1.41 | -1.12 |
| Qatar | -1.60 | -1.79 | -1.41 |
| Republic of Korea | -5.48 | -5.67 | -5.28 |
| Republic of Moldova | -0.35 | -1.01 | 0.32 |
| Romania | -2.25 | -2.55 | -1.94 |
| Russian Federation | -1.48 | -2.02 | -0.92 |
| Rwanda | -1.77 | -2.18 | -1.36 |
| Saint Kitts and Navis | -1.85 | -1.99 | -1.71 |
| Saint Lucia | -1.35 | -1.41 | -1.29 |
| Saint Vincent and the Grenadines | -2.08 | -2.18 | -1.97 |
| Samoa | -1.04 | -1.14 | -0.93 |
| San Marino | -0.83 | -1.17 | -0.49 |
| Sao Tome and Principe | -1.06 | -1.28 | -0.84 |
| Saudi Arab Republic | -1.17 | -1.34 | -0.99 |
| Scotland | -1.15 | -1.24 | -1.06 |
| Senegal | -0.70 | -0.78 | -0.61 |
| Serbia | -1.10 | -1.36 | -0.83 |
| Seychelles | -2.11 | -2.21 | -2.00 |
| Sierra Leone | 0.11 | -0.27 | 0.49 |
| Singapore | -4.51 | -4.78 | -4.23 |
| Slovakia | -2.39 | -2.62 | -2.16 |
| Slovenia | -2.07 | -2.26 | -1.88 |
| Solomon Islands | -0.01 | -0.16 | 0.14 |
| Somalia | 0.66 | 0.48 | 0.84 |
| South Africa | -1.07 | -1.20 | -0.93 |
| South Sudan | -0.65 | -0.85 | -0.45 |
| Spain | -1.33 | -1.46 | -1.20 |
| Sri Lanka | -3.24 | -3.41 | -3.07 |
| Suriname | -1.58 | -1.76 | -1.40 |
| Sweden | -0.83 | -0.97 | -0.68 |
| Switzerland | -0.82 | -0.90 | -0.75 |
| Taiwan (Province of China) | -3.06 | -3.19 | -2.92 |
| Tajikistan | 0.31 | -0.38 | 1.01 |
| Thailand | -3.17 | -3.26 | -3.08 |
| Timor-Leste | -2.92 | -3.23 | -2.61 |
| Togo | -0.08 | -0.26 | 0.11 |
| Tokelau | -1.71 | -1.82 | -1.60 |
| Tonga | -0.63 | -0.68 | -0.58 |
| Trinidad and Tobago | -2.82 | -3.07 | -2.57 |
| Tunisia | -1.77 | -1.81 | -1.73 |
| Turkey | -2.09 | -2.20 | -1.99 |
| Turkmenistan | -2.58 | -3.26 | -1.89 |
| Tuvalu | -0.90 | -1.04 | -0.76 |
| Uganda | -1.99 | -2.10 | -1.88 |
| Ukraine | -0.34 | -0.87 | 0.19 |
| United Arab Emirates | 0.74 | 0.55 | 0.94 |
| United Republic of Tanzania | -1.75 | -1.98 | -1.51 |
| United States of America | -0.93 | -1.00 | -0.85 |
| United States Virgin Islands | -2.06 | -2.42 | -1.70 |
| Uruguay | -1.36 | -1.53 | -1.20 |
| Uzbekistan | -1.55 | -1.94 | -1.15 |
| Vanuatu | -0.27 | -0.30 | -0.24 |
| Viet Nam | -3.43 | -3.53 | -3.34 |
| Wales | -1.79 | -1.90 | -1.68 |
| Yemen | -0.63 | -0.90 | -0.37 |
| Zambia | -1.58 | -2.01 | -1.15 |
| Zimbabwe | 1.44 | 1.08 | 1.81 |
