# Supplementary material for: Significant regional inequalities in the prevalence of intellectual disability and trends from 1990 to 2019: a systematic analysis of GBD 2019
Source: Epidemiol Psychiatr Sci. 2022 Dec 21;31:e91. doi: 10.1017/S2045796022000701 (PMC9805697; doi:10.1017/S2045796022000701)
Supplement: Supplementary file 1 [file epssup.zip › S2045796022000701sup003.docx]

**R codes used in the study**

install.packages("psych")

library(psych)

#calculate APC for low SDI countries over 30 years by age ranges

rate.low <- read.csv(file.choose())

rate.low.1 <- subset(rate.low, age_name=="0 to 9", select = c(val, year))

model.1 <- lm(log(val)~year, rate.low.1)

summary(model.1)

apc <- 100*(exp(model.1$coefficients[2])-1)

apc.lower <- 100*(exp(model.1$coefficients[2]-1.96*coef(summary(model.1))[2,2])-1)

apc.upper <- 100*(exp(model.1$coefficients[2]+1.96*coef(summary(model.1))[2,2])-1)

apc

apc.lower

apc.upper

rate.low.2 <- subset(rate.low, age_name=="10 to 24", select = c(val, year))

model.2 <- lm(log(val)~year, rate.low.2)

summary(model.2)

apc <- 100*(exp(model.2$coefficients[2])-1)

apc.lower <- 100*(exp(model.2$coefficients[2]-1.96*coef(summary(model.2))[2,2])-1)

apc.upper <- 100*(exp(model.2$coefficients[2]+1.96*coef(summary(model.2))[2,2])-1)

apc

apc.lower

apc.upper

rate.low.3 <- subset(rate.low, age_name=="25 to 49", select = c(val, year))

model.3 <- lm(log(val)~year, rate.low.3)

summary(model.3)

apc <- 100*(exp(model.3$coefficients[2])-1)

apc.lower <- 100*(exp(model.3$coefficients[2]-1.96*coef(summary(model.3))[2,2])-1)

apc.upper <- 100*(exp(model.3$coefficients[2]+1.96*coef(summary(model.3))[2,2])-1)

apc

apc.lower

apc.upper

rate.low.4 <- subset(rate.low, age_name=="50-69 years", select = c(val, year))

model.4 <- lm(log(val)~year, rate.low.4)

summary(model.4)

apc <- 100*(exp(model.4$coefficients[2])-1)

apc.lower <- 100*(exp(model.4$coefficients[2]-1.96*coef(summary(model.4))[2,2])-1)

apc.upper <- 100*(exp(model.4$coefficients[2]+1.96*coef(summary(model.4))[2,2])-1)

apc

apc.lower

apc.upper

rate.low.5 <- subset(rate.low, age_name=="70+ years", select = c(val, year))

model.5 <- lm(log(val)~year, rate.low.5)

summary(model.5)

apc <- 100*(exp(model.5$coefficients[2])-1)

apc.lower <- 100*(exp(model.5$coefficients[2]-1.96*coef(summary(model.5))[2,2])-1)

apc.upper <- 100*(exp(model.5$coefficients[2]+1.96*coef(summary(model.5))[2,2])-1)

apc

apc.lower

apc.upper

###calculate APC value for each country

rate <- read.csv(file.choose())

model <- lm(log(val)~year, rate)

summary(model)

apc <- 100*(exp(model$coefficients[2])-1)

apc

# Install Libraries for plotting inequality results

install.packages("ggplot2")

library(ggplot2)

install.packages("ggpubr")

library(ggpubr)

installed.packages("psych")

library(psych)

# read data

ai <- read.csv(file.choose())

# plot1: absolute inequalities

aigraph <- ggplot(ai, aes(year, AI, group = age, color = age)) + geom_line() + geom_point() + scale_y_continuous(expand = c(0,0),limits = c(0, 3000), breaks = seq(0, 3000, by = 500))+

scale_x_continuous(limits=c(1990,2019),breaks = seq(1990, 2019, by = 4)) +ggtitle("Absolute Inequalities") + xlab("Year") + ylab("Absolute Inequalities")+

theme(axis.text=element_text(size=15))+theme(axis.title=element_text(size=18))+theme(legend.text=element_text(size=12))+theme(plot.title=element_text(size=20))+theme(legend.position="bottom")

# plot2: relative inequalities

rigraph <- ggplot(ai, aes(year, RI, group = age, color = age)) + geom_line() + geom_point() + scale_y_continuous(expand=c(0,0),limits = c(0, 7.5), breaks = seq(0, 7.5, by = 1))+

scale_x_continuous(limits=c(1990,2019),breaks = seq(1990, 2019, by = 4)) +ggtitle("Relative Inequalities") + xlab("Year") + ylab("Relative Inequalities")+

theme(axis.text=element_text(size=15))+theme(axis.title=element_text(size=18))+theme(legend.text=element_text(size=12))+theme(plot.title=element_text(size=20))+theme(legend.position="bottom")

# combine two graphs in one

ggarrange(aigraph, rigraph, labels = c("A", "B"), ncol= 2, nrow = 1)

#create graphs for different SDIs

ai <- read.csv(file.choose())

newai <- ai[c(-2,-3)]

#subset data by age groups

newai_0 <- subset(newai, age == "0-9 years" )

newai_10 <- subset(newai, age == "10-24 years")

newai_25 <- subset(newai, age == "25-49 years")

newai_50 <- subset(newai, age == "50-69 years")

newai_70 <- subset(newai, age == "70+ years")

newai_0 = newai_0[c(-2)]

newai_10 = newai_10[c(-2)]

newai_25 = newai_25[c(-2)]

newai_50 = newai_50[c(-2)]

newai_70 = newai_70[c(-2)]

#convert wide form into long form for each age group

install.packages("reshape2")

library(reshape2)

newai_0long <- melt(newai_0, id.vars=c("year"), measure.vars = c("Low.SDI", "Low.Middle.SDI","Middle.SDI","High.Middle.SDI","High.SDI"),

variable.name = "SDI", value.name="AI")

newai_10long <- melt(newai_10, id.vars=c("year"), measure.vars = c("Low.SDI", "Low.Middle.SDI","Middle.SDI","High.Middle.SDI","High.SDI"),

variable.name = "SDI", value.name="AI")

newai_25long <- melt(newai_25, id.vars=c("year"), measure.vars = c("Low.SDI", "Low.Middle.SDI","Middle.SDI","High.Middle.SDI","High.SDI"),

variable.name = "SDI", value.name="AI")

newai_50long <- melt(newai_50, id.vars=c("year"), measure.vars = c("Low.SDI", "Low.Middle.SDI","Middle.SDI","High.Middle.SDI","High.SDI"),

variable.name = "SDI", value.name="AI")

newai_70long <- melt(newai_70, id.vars=c("year"), measure.vars = c("Low.SDI", "Low.Middle.SDI","Middle.SDI","High.Middle.SDI","High.SDI"),

variable.name = "SDI", value.name="AI")

#plot for each age group

newai_0graph <- ggplot(newai_0long, aes(year, AI, group = SDI, color = SDI)) + geom_line() + geom_point() + scale_y_continuous(expand = c(0,0),limits = c(0, 4500), breaks = seq(0, 4500, by = 500))+

scale_x_continuous(limits=c(1990,2019),breaks = seq(1990, 2019, by = 4)) +ggtitle("0-9 years") + ylab("Absolute Inequalities") + xlab(NULL)+

theme(axis.text=element_text(size=10))+theme(axis.title=element_text(size=18))+theme(legend.text=element_text(size=12))+theme(plot.title=element_text(size=20))+theme(legend.position="none")

newai_10graph <- ggplot(newai_10long, aes(year, AI, group = SDI, color = SDI)) + geom_line() + geom_point() + scale_y_continuous(expand = c(0,0),limits = c(0, 4500), breaks = seq(0, 4500, by = 500))+

scale_x_continuous(limits=c(1990,2019),breaks = seq(1990, 2019, by = 4)) +ggtitle("10-24 years")+ ylab(NULL) + xlab(NULL)+

theme(axis.text=element_text(size=10))+theme(axis.title=element_text(size=18))+theme(legend.text=element_text(size=12))+theme(plot.title=element_text(size=20))+theme(legend.position="none")

newai_25graph <- ggplot(newai_25long, aes(year, AI, group = SDI, color = SDI)) + geom_line() + geom_point() + scale_y_continuous(expand = c(0,0),limits = c(0, 4500), breaks = seq(0, 4500, by = 500))+

scale_x_continuous(limits=c(1990,2019),breaks = seq(1990, 2019, by = 4)) +ggtitle("25-49 years") + ylab("Absolute Inequalities")+ xlab(NULL)+

theme(axis.text=element_text(size=10))+theme(axis.title=element_text(size=18))+theme(legend.text=element_text(size=12))+theme(plot.title=element_text(size=20))+theme(legend.position="none")

newai_50graph <- ggplot(newai_50long, aes(year, AI, group = SDI, color = SDI)) + geom_line() + geom_point() + scale_y_continuous(expand = c(0,0),limits = c(0, 4500), breaks = seq(0, 4500, by = 500))+

scale_x_continuous(limits=c(1990,2019),breaks = seq(1990, 2019, by = 4)) +ggtitle("50-69 years")+ ylab(NULL) + xlab("Year") +

theme(axis.text=element_text(size=10))+theme(axis.title=element_text(size=18))+theme(legend.text=element_text(size=12))+theme(plot.title=element_text(size=20))+theme(legend.position="none")

newai_70graph <- ggplot(newai_70long, aes(year, AI, group = SDI, color = SDI)) + geom_line() + geom_point() + scale_y_continuous(expand = c(0,0),limits = c(0, 4500), breaks = seq(0, 4500, by = 500))+

scale_x_continuous(limits=c(1990,2019),breaks = seq(1990, 2019, by = 4)) +ggtitle("70+ years")+ ylab("Absolute Inequalities") + xlab("Year") +

theme(axis.text=element_text(size=10))+theme(axis.title=element_text(size=18))+theme(legend.text=element_text(size=20))+theme(plot.title=element_text(size=20))+theme(legend.position="bottom")

# combine five graphs in one

ggarrange(newai_0graph, newai_10graph, newai_25graph, newai_50graph, newai_70graph, labels = c("A", "B", "C", "D", "E"),ncol= 2, nrow = 3)
